# Paenilamicins are context-specific translocation inhibitors of protein synthesis

**DOI:** 10.1038/s41589-024-01752-9

**Published:** 2024-10-17

**Authors:** Timm O. Koller, Max J. Berger, Martino Morici, Helge Paternoga, Timur Bulatov, Adriana Di Stasi, Tam Dang, Andi Mainz, Karoline Raulf, Caillan Crowe-McAuliffe, Marco Scocchi, Mario Mardirossian, Bertrand Beckert, Nora Vázquez-Laslop, Alexander S. Mankin, Roderich D. Süssmuth, Daniel N. Wilson

**Affiliations:** 1 Institute for Biochemistry and Molecular Biology, University of Hamburg, Hamburg, Germany; 2 Institut für Chemie, Technische Universität Berlin, Berlin, Germany; 3 Department of Life Sciences, University of Trieste, Trieste, Italy; 4 Dubochet Center for Imaging (DCI) at EPFL, EPFL SB IPHYS DCI, Lausanne, Switzerland; 5 Center for Biomolecular Sciences, University of Illinois at Chicago, Chicago, IL USA; 6 Department of Pharmaceutical Sciences, University of Illinois at Chicago, Chicago, IL USA

**Keywords:** Structural biology, Translation, Small molecules, RNA, Natural products

## Abstract

The paenilamicins are a group of hybrid nonribosomal peptide–polyketide compounds produced by the honey bee pathogen *Paenibacillus larvae* that display activity against Gram-positive pathogens, such as *Staphylococcus aureus*. While paenilamicins have been shown to inhibit protein synthesis, their mechanism of action has remained unclear. Here we determine structures of paenilamicin PamB2-stalled ribosomes, revealing a unique binding site on the small 30S subunit located between the A- and P-site transfer RNAs (tRNAs). In addition to providing a precise description of interactions of PamB2 with the ribosome, the structures also rationalize the resistance mechanisms used by *P. larvae*. We further demonstrate that PamB2 interferes with the translocation of messenger RNA and tRNAs through the ribosome during translation elongation, and that this inhibitory activity is influenced by the presence of modifications at position 37 of the A-site tRNA. Collectively, our study defines the paenilamicins as a class of context-specific translocation inhibitors.

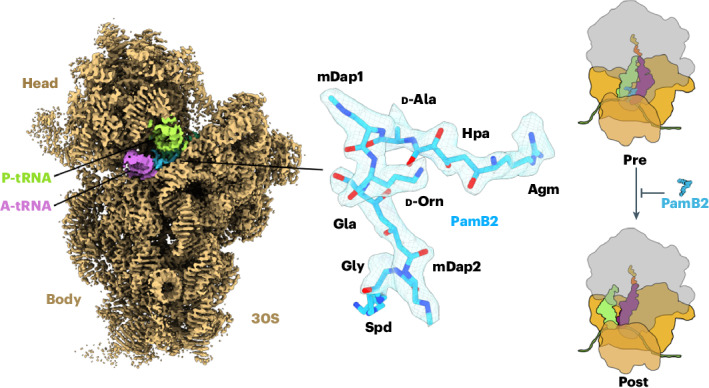

## Abstract (web summary)

The paenilamicins are hybrid nonribosomal peptide–polyketide compounds that inhibit protein synthesis. Here the authors reveal that paenilamicins bind to a unique site on the ribosome, where they interfere with the translocation of mRNA and tRNAs during elongation.

## Main

The increase in multi-drug resistance is making our current arsenal of clinically relevant antibiotics obsolete^1^, with a notable contribution coming from so-called ESKAPE pathogens^2^. This problem is compounded by the rapid decline in the approval of new antibiotics, particularly those with novel scaffolds^1^, highlighting the need for the discovery and development of further antimicrobials. One potential class are the paenilamicins, a group of hybrid nonribosomal peptide–polyketide compounds that display activity against Gram-positive bacteria, such as *Staphylococcus aureus*^3,4^. Paenilamicins were shown to be fourfold more active against methicillin-resistant *S. aureus* than the gold-standard ciprofloxacin^4^, and also display activity against the opportunistic fungal pathogens *Sporobolomyces salmonicolor* and *Aspergillus fumigatus*^4^. Paenilamicins are produced by the bacterium *Paenibacillus larvae*, which is the causative agent of American Foulbrood: the most destructive bacterial brood disease affecting honey bees world-wide^5^. Infection assays using bee larvae and the insect pathogen *Bacillus thuringiensis* demonstrated that paenilamicin production by *P. larvae* is used for suppression of bacterial competitors during host infection^4^.

**Fig. 1:**
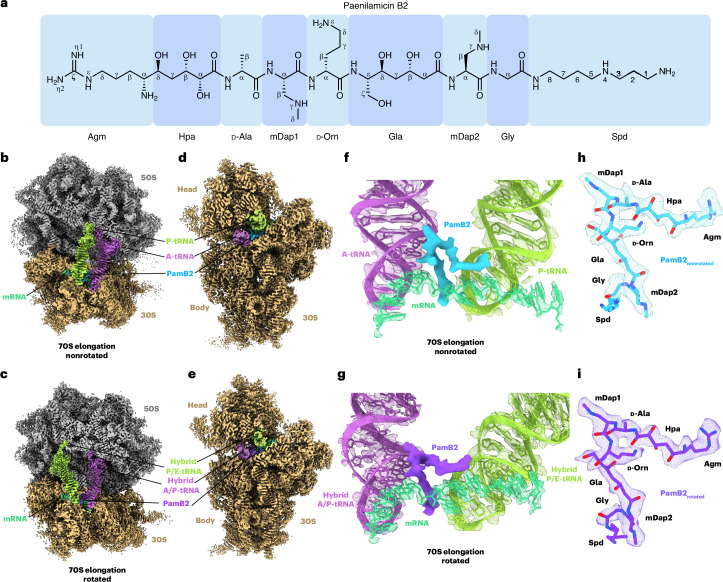
Cryo-EM structures of paenilamicin B2 on the ribosome. **a**, Chemical structure of paenilamicin B2 (refs. ^3,4^). **b**–**e**, Cryo-EM map of the PamB2-stalled ribosome in nonrotated (**b**,**d**) and rotated (**c**,**e**) elongation state, shown as transverse section (**b,c**) and 20S interface view (**d,e**). **f**,**g**, Extracted cryo-EM density assigned to PamB2 from the nonrotated (**f**, light blue) and rotated (**g**, dark purple) with surrounding A-tRNA (purple), P-tRNA (light green) from the nonrotated (**f**) and hybrid A/P (purple), and P/E-tRNA (light green) (**g**) and mRNA (cyan) in extracted density shown as mesh. **h**,**i**, Molecular model of PamB2 in extracted density of the nonrotated (**h**, light blue) and rotated (**i**, dark purple) states, shown as a mesh.

**Extended Data Fig. 1:**
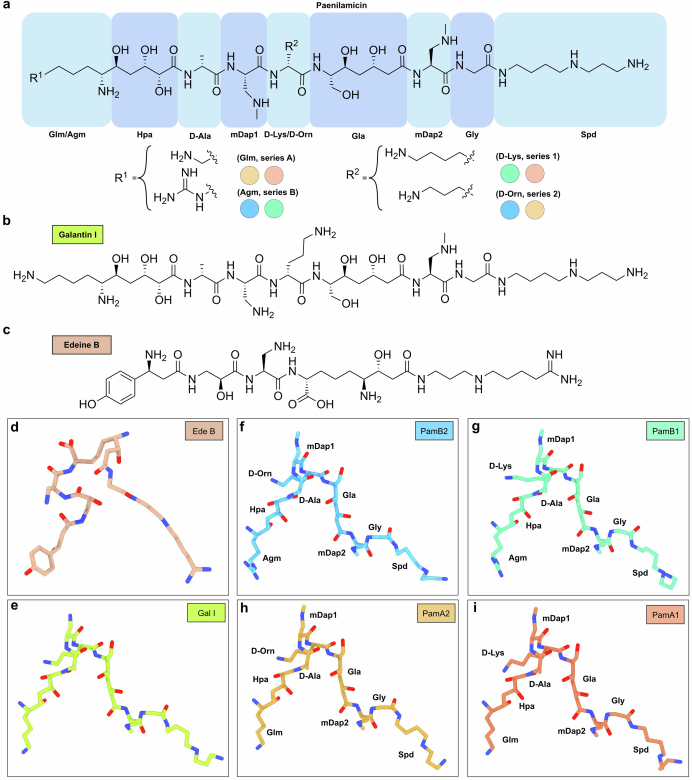
Chemical structures and models of paenilamicin, galantin I and edeine. **a–c**, Chemical structures of (**a**) paenilamicin, (**b**) galantin I and (**c**) edeine B. **d**, Molecular model of edeine B on the *T. thermophilus* 30S subunit (beige, Ede B, PDB ID 1I95)^42^. **e**, *In silico* molecular model of galantin I (Gal I, light green). **f-i**, Molecular model of PamB2 of the non-rotated PamB2 complex and *in silico* modelled PamB1 (green), PamA2 (light orange), PamA1 (orange).

To date, four distinct paenilamicins have been structurally elucidated, PamA1, PamA2, PamB1 and PamB2 (Fig. 1a and Extended Data Fig. 1a)^3,4^. The d-Agm of PamB1 and PamB2 is substituted for cadaverine in PamA1 and PamA2, respectively^3^ (Extended Data Fig. 1a). The structure of the paenilamicins is closely related to that of galantin I (Extended Data Fig. 1b), a compound originally isolated from a soil bacterium from New Guinea in the 1970s^6^. A recent structural revision of PamB2 revealed that the (α*R*)-configuration of the terminal amino group in Agm is important for maximal activity and plays a role in self-resistance, being required for acetylation by PamZ and, thereby, inactivation of the drug^4,7^. The protection of the N-terminus during biosynthesis by attachment of an *N*-acyl-d-Asn moiety is also a prominent prodrug resistance mechanism^8^.

The accordance of structural features of paenilamicins with the translation inhibitor edeine (Extended Data Fig. 1c) led to the hypothesis that paenilamicins may also exert their antimicrobial activity by binding to the ribosome and inhibiting protein synthesis^4^. In this regard, PamB2 was shown to be a potent inhibitor of *Escherichia coli* in vitro translation, with a half-inhibitory concentration (IC_50_) of 0.4 μM (ref. ^4^), lower than that reported for the well-known translation inhibitors erythromycin, chloramphenicol and tetracycline that display IC_50_ values of 0.75, 1.0 or 10 μM, respectively^9–11^. Although PamB2_2, which contains the nonnative (α*S*)-configuration, retained inhibitory activity in the *E. coli* in vitro translation systems, a 7–10-fold reduction in efficiency (IC_50_ of 2.9 μM) was observed^4^. More dramatic was the effect of the PamZ-mediated acetylation of the α-amino group of Agm in PamB2, which increased the IC_50_ by almost 80-fold to 31.9 μM (ref. ^4^). This suggests that the acetylation of PamB2 may interfere with the binding of the compound to the target. However, whether the ribosome is indeed the target, and how PamB2 inhibits protein synthesis remains to be elucidated.

Here we have used single-particle cryogenic electron microscopy (cryo-EM) to determine structures of ribosomes stalled during translation by the binding of PamB2. The structures, refined to 2.2–2.4 Å resolution, reveal that PamB2 binds stably to the small subunit of elongating ribosomes containing A- and P-site transfer RNAs (tRNAs), but not to initiating ribosomes bearing only a P-site tRNA, indicating that the presence of A-site tRNA is critical for PamB2 binding. This binding site of PamB2 is distinct from any other antibiotic binding site on the 30S subunit being located between the A- and P-site tRNAs. The structures also rationalize the increased activity of the native (α*R*)-configuration as well as the mechanism of self-resistance used by the producer. Biochemical data demonstrate that PamB2 inhibits the EF-G catalyzed translocation step of protein synthesis in a highly context-specific manner that is dependent on the type of modifications that are present at position 37 of the A-site tRNA. Thus, paenilamicins represent a class of context-specific translocation inhibitors that are influenced by the modification state of the tRNA.

## Results

### Cryo-EM structures of PamB2 on the ribosome

**Extended Data Fig. 2:**
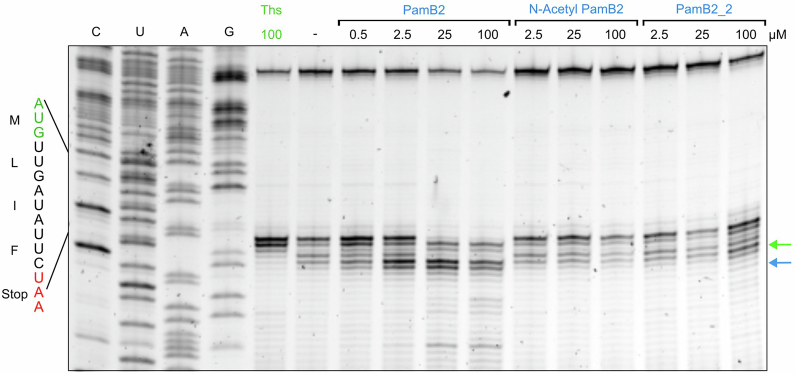
Toeprinting assay on the MLIFstop-mRNA. Toeprinting assay monitoring the position of ribosomes on a MLIFstop-mRNA in the presence of 100 µM thiostrepton (Ths), water (–), increasing concentrations of PamB2, N-Acetyl-PamB2 and PamB2_2 (0.5-100 µM). Arrows indicate the stalling at the initiation codon (green), and PamB2 induced stalling (blue). Toeprinting assays were performed in duplicate, with the duplicate gel shown in the Source Data. Source data

To investigate how paenilamicins inhibit translation, we generated PamB2-stalled ribosome complexes (PamB2-SRC) for single-particle cryo-EM analysis. Rather than forming complexes on vacant ribosomes, or with predefined functional states, we instead aimed to use more physiological complexes where translating ribosomes become stalled by PamB2. To achieve this, we performed in vitro translation reactions with *E. coli* ribosomes using the Met-Leu-Ile-Phe-stop-mRNA (MLIFstop-mRNA), a model template that we had previously used to successfully determine structures of drosocin-stalled ribosomal complexes^12^. Toeprinting was used to monitor the position of ribosomes on the MLIFstop-mRNA in the presence of increasing concentrations of synthetic PamB2 (Extended Data Fig. 2). As a positive control, we used thiostrepton that traps ribosomes on the AUG initiation codon in cell-free systems^13–15^, and as a negative control, we included the inactive *N*-acetylated form of PamB2 (*N*-Ac-PamB2)^4^ (Extended Data Fig. 2). In the absence of drug, bands are evident for ribosomes on the AUG start codon and the adjacent UUG (Leu) codon, suggesting that initiation and/or the first two elongation steps are slow on this messenger RNA (mRNA) or that the mRNA contains secondary structure in this region. In the presence of thiostrepton, a single strong band is observed that corresponds to ribosomes trapped on the AUG start codon (Extended Data Fig. 2), as expected^13–15^. By contrast, increasing concentrations of PamB2 led to a gradual loss of ribosomes at the AUG codon and an increase in ribosomes stalled one codon further with the UUG (Leu) codon in the P-site. This shift in ribosome positioning was not observed for the *N*-Ac-PamB2, where the pattern looks similar to the no-drug control, consistent with the inactivity of this compound^4^ (Extended Data Fig. 2). We also tested PamB2_2 with the nonnative (α*S*)-configuration that, like *N*-Ac-PamB2, appeared to have little inhibitory activity in this assay (Extended Data Fig. 2).

**Extended Data Fig. 3:**
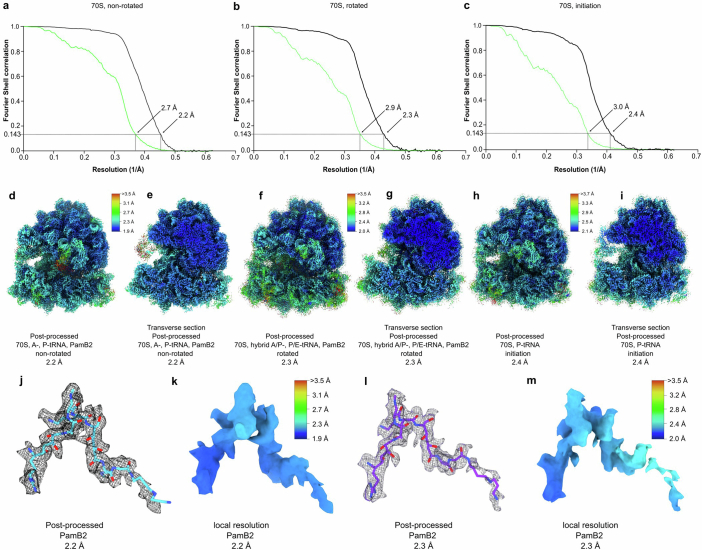
Fourier shell correlation and local resolution for the PamB2 complexes. **a–c**, Fourier shell correlation (FSC) curve of the (**a**) non-rotated, (**b**) rotated and (**c**) initiation complexes, with unmasked (green) and masked (black) FSC curves plotted against the resolution (1/Å). **d–i**, Cryo-EM density colored according to local resolution and transverse section for the (**d, e**) non-rotated, (**f, g**) rotated and (**h, i**) initiation complexes. **j–m**, Molecular model of PamB2 (light blue and purple) and corresponding cryo-EM density colored according to local resolution for the (**j, k**) non-rotated, and (**l–m**) rotated complex.

PamB2-SRCs were generated as above using 50 μM PamB2 and subjected to single-particle cryo-EM analysis. In silico sorting of the cryo-EM data revealed three main populations of ribosomal states, namely nonrotated 70S ribosomes with P-site tRNA only (15%), or with A- and P-site tRNAs (31%), as well as a population containing rotated 70S ribosomes with A/P- and P/E-hybrid site tRNAs (17 %) (Supplementary Fig. 1), which after refinement yielded final reconstructions with average resolutions of 2.4, 2.2 and 2.3 Å, respectively (Fig. 1b–e, Extended Data Fig. 3a–I, Supplementary Video 1 and Supplementary Table 1). In both reconstructions containing two tRNAs, we observed additional density located between the A- and P-site tRNAs that could be unambiguously assigned to PamB2 (Fig. 1f–i). The density of PamB2 was well-resolved, enabling the orientation of the inhibitor to be determined, and the N-terminal Agm and Hpa as well as central d-Ala, d-Orn, mDap1 and mDap2 and Gla moieties to be modeled (Fig. 1h,i and Supplementary Fig. 2). The exception was the C-terminal Spd moiety that was poorly ordered in both maps, with density observed only at low thresholds (Extended Data Fig. 3j–m). No density for PamB2 was evident in the cryo-EM reconstruction where only one tRNA (the initiator tRNA in the P-site) was present, suggesting that PamB2 may require an A-site tRNA to bind stably to the ribosome.

### Interaction of PamB2 with the ribosomal P-site

**Fig. 2:**
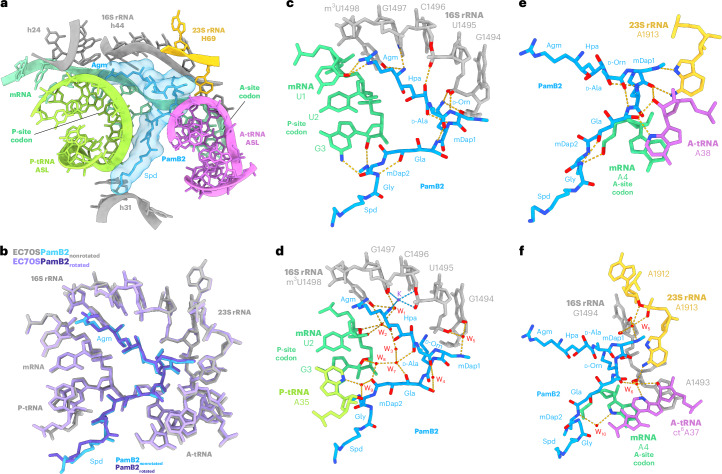
Interaction of PamB2 with the ribosomal P- and A-sites. **a**, PamB2 (light blue) binding pocket located on the 30S subunit of the nonrotated PamB2–70S complex, with A-site tRNA (purple), P-site tRNA (light green), 16S rRNA (gray), 23S rRNA (yellow) and mRNA (cyan). **b**, Superimposition of the PamB2 binding pocket of the nonrotated (gray, with PamB2 in light blue) and rotated (light purple with PamB2 in purple) PamB2–70S complexes. **c**–**f**, Direct and water-mediated interactions (dashed yellow lines) between PamB2 and the ribosome, colored as in **a**. **c**, Direct and intramolecular interactions of PamB2 with 16S rRNA of h44 and mRNA of the P-site codon. **d**, Water-mediated interactions of PamB2 with h44 of the 16S rRNA, mRNA of the P-site codon and P-site tRNA. **e**, Direct and intramolecular interactions of PamB2 with H69 of the 23S rRNA, mRNA of the A-site codon and A-site tRNA. **f**, Water-mediated interactions of PamB2 with h44 of the 16S rRNA, H69 of the 23S rRNA, mRNA of the A-site codon and A-site tRNA.

The PamB2 binding site is located predominantly on the 30S subunit of the 70S ribosome, where it inserts into the cleft between the A- and P-site tRNAs (Fig. 2a, Supplementary Fig. 2 and Supplementary Video 1). Although we describe the interactions of PamB2 for the nonrotated A- and P-site tRNA state, we note that within the limits of the resolution of the reconstructions, the binding mode of PamB2 is similar, if not identical, for the rotated A/P- and P/E-hybrid state (Fig. 2b). In both states, PamB2 is oriented with the Agm side chain extending toward h24, while the central region of PamB2 runs parallel to the mRNA as well as one strand of nucleotides in h44 (Fig. 2a). The central mDap1 region of PamB2 interacts with H69 of the 23S rRNA, and then kinks such that the C-terminal (Gla-mDap2-Gly-Spd) region passes between the A- and P-site tRNAs, with the Spd moiety extending toward h31 (Fig. 2a). The kinked conformation of PamB2 is likely to be stabilized by three intramolecular hydrogen bonds (Fig. 2c), as well as two water-mediated interactions (Fig. 2d). The structural similarity with PamB2 (Extended Data Fig. 1d–i) suggests other paenilamicins (PamB1, PamA1 and PamA2) and also galantin I are likely to interact with the ribosome in the same manner.

**Extended Data Fig. 4:**
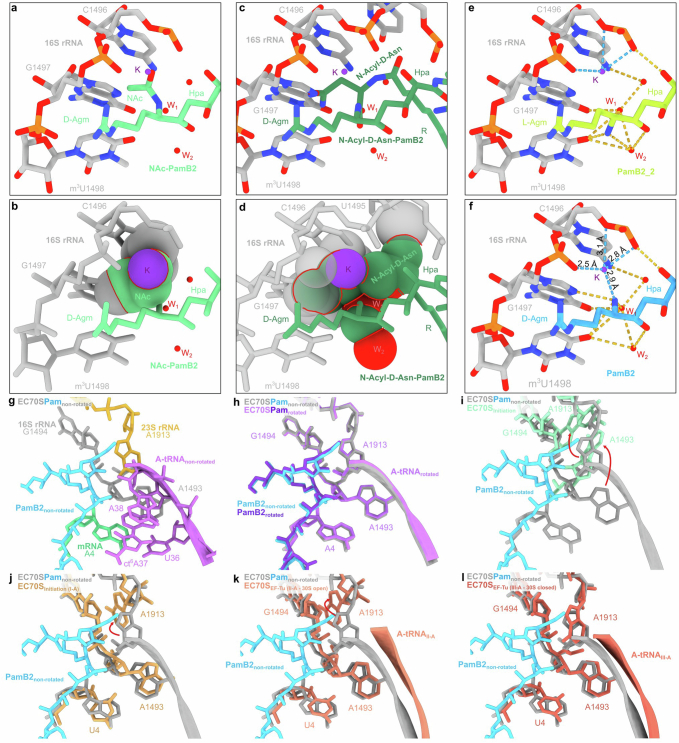
PamB2 *in silico* modification and A-site binding pocket. **a**–**d**, *In silico* model of (**a, b**) N-acetyl-PamB2 (green) shown as (**a**) stick and (**b**) sphere representation, and (**c, d**) PamB2 with an *N*-acyl-D-Asn moiety (dark green) shown as (**c**) stick and (**d**) sphere representation. In (**b**) and (**d**), the sterically clashing molecules are highlighted by red lines. **e**, Interactions of the *in silico* modified PamB2_2 (light green) L-Agm amino group with surrounding 16S rRNA nucleotides of h44. **f**, Interactions of the PamB2 (light blue) wildtype D-Agm amino group with surrounding 16S rRNA nucleotides of h44. **g**, Binding pocket of PamB2 (light blue) with surrounding A-site tRNA nucleotides (purple), 16S rRNA (grey), mRNA (cyan) and 23S rRNA (yellow) superimposed with (**h**) the rotated PamB2 complex (purple), (**i**) the initiation complex (light cyan), (**j**) the *E. coli* initiation state (yellow, I-A, PDB ID 6WD0)^18^, (**k**) EF-Tu bound to the open *E. coli* 30S subunit (orange, II-A, PDB-ID 6WD2)^18^, and (**l**) EF-Tu bound to the closed *E. coli* 30S subunit (dark orange, III-A, PDB-ID 6WD8)^18^.

In the P-site, most of the interactions of PamB2 are with 16S rRNA nucleotides (G1494-m^3^U1498) in h44, on one side and with the P-site codon of the mRNA on the other (Fig. 2c,d). Together with U1495, C1496 and G1497, the N-terminal amino group of Agm coordinates an ion, which we assign to a K^+^ ion based on the coordination distances and the presence of a K^+^ ion in a similar position of a previous *E. coli* 70S–hygromycin B structure^16^. We note that acetylation of the N-terminal amino group of Agm by the *N*-acetyltransferase PamZ^4,7^, or modification with *N*-acyl-d-Asn^8^, inactivates PamB2. Modeling these modified forms of PamB2 into the binding site indicates that they would clash with the surrounding 16S rRNA (Extended Data Fig. 4a–d), suggesting that these modifications would prevent PamB2 from binding to the ribosome. The binding mode of PamB2 also explains the reduction in activity of PamB2_2 since the (α*S*)-configuration of the N-terminal amino group of Agm would lead to loss of direct contact with the N7 of G1497, as well as the K^+^ ion-mediated interaction with 16S rRNA nucleotides in h44 (Extended Data Fig. 4e,f).

With regard to the P-site codon of the mRNA, there are two main points of contact, namely, the O2 of U1 (first position of the P-site codon) with the η2- and ε-nitrogens of Agm (Fig. 2c), and second, involving G3, located in the third position of the codon, where the ribose O2′ and N2 can form hydrogen bonds with γ-nitrogen and carbonyl–oxygen of mDap2 of PamB2 (Fig. 2c). In addition, water molecules (W_2_ and W_8_) mediate interactions between the backbone of U2 and Hpa of PamB2, as well as the O4′ (ribose) of G3 with the carbonyl–oxygen of Gla of PamB2 (Fig. 2d). Although PamB2 approaches the P-site tRNA, there is relatively little direct interaction, with the closest point of contact being 3.6 Å between the η2-nitrogen of Agm and the ribose O2′ of A37 of the P-site tRNA. However, we do observe a water-mediated (W_9_) interaction between the carbonyl–oxygen of Gla of PamB2 and the O2′ and N3 of A35 of the P-site tRNA (Fig. 2d).

### Interaction of PamB2 with the ribosomal A-site

**Extended Data Fig. 5:**
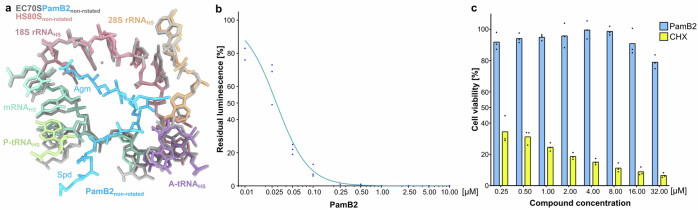
PamB2 inhibits eukaryotic translation. **a**, Superimposition of the PamB2 (light blue) binding site on bacterial ribosomes (grey) with that of the eukaryotic ribosome (PDB ID 6Y0G)^59^ with 18S rRNA nucleotides (pink), 28S rRNA nucleotides (light orange), P-tRNA (light green), A-tRNA (purple) and mRNA (light cyan), illustrating the high conservation. **b**, In vitro translation assay using rabbit reticulocyte lysate-based system to monitor inhibition of increasing concentrations of PamB2. Reactions were performed in triplicate and the line of best fit is shown. **c**, MTT assay on NIH 3T3 murine fibroblast cells after treatment with different concentrations of PamB2 and CHX. The cell viability was measured (as absorbance at 570 nm) after 48 h of exposure to the peptides. Results are reported as percentages of viable cells with respect to the negative control cells with no compound (set as 100% of viability). Data are the average of three biological replicates, each of which was performed with internal triplicates and averaged. The individual values are available in the Source Data. Source data

In the A-site, PamB2 contacts not only with 16S rRNA nucleotides in h44, but also A1913 from H69 of the 23S rRNA, and, in contrast to the P-site, the compound makes extensive interactions with the A-site tRNA, albeit less with the mRNA codon (Fig. 2c–f). Interactions of PamB2 with the A-site tRNA revolve around nucleotides ct^6^A37 and A38, which are located in the anticodon-stem loop, directly adjacent to the anticodon (_34_CAU_36_) (Fig. 2e,f). Specifically, three direct hydrogen bonds are possible with A38 (Fig. 2e). Interactions with ct^6^A37 are indirect, being mediated by water W_6_, which is coordinated by the carbonyl–oxygen of D-Orn as well as the O2′ and N3 of ct^6^A37 (Fig. 2f). Interaction of PamB2 with the A-site codon of the mRNA is restricted to a direct interaction of backbone amide of Gly and a backbone oxygen of A4, which is located in the first position of the A-site codon, and a water-mediated interaction from the backbone amide of mDap2 via water W_10_ with N7 (3.4 Å) of A4 (Fig. 2f). The interactions between PamB2 and the A-site tRNA are likely to be critical for binding of PamB2 to the ribosome, since we observe no density for PamB2 in the P-tRNA-only state. We note that in structures of 70S ribosomes lacking A-tRNA^17^, the conformation of A1913 in H69 differs from that when the A-site tRNA is present, such that it would be incompatible with the interactions observed for PamB2 on the elongating ribosome (Extended Data Fig. 4g–j). The A1913 conformational shift induced by A-tRNA binding may therefore contribute to preventing stable binding of PamB2. Although a shift in the position of A1913 occurs during decoding when the A-site tRNA is still bound to EF-Tu, the A-tRNA itself is still suboptimally placed to interact with PamB2 in such a state^18^ (Extended Data Fig. 4k,l), suggesting that full accommodation of A-tRNA is required for stable interaction of PamB2 with the ribosome. We note that the PamB2 binding site is conserved on eukaryotic ribosomes (Extended Data Fig. 5a), and could accordingly demonstrate that PamB2 efficiently inhibits eukaryotic in vitro translation (Extended Data Fig. 5b), consistent with its antifungal activity^4^. However, PamB2 displays no cytotoxicity against eukaryotic cell lines (Extended Data Fig. 5c), suggesting that the compound is not internalized.

### PamB2 inhibits tRNA_2_–mRNA translocation

**Fig. 3:**
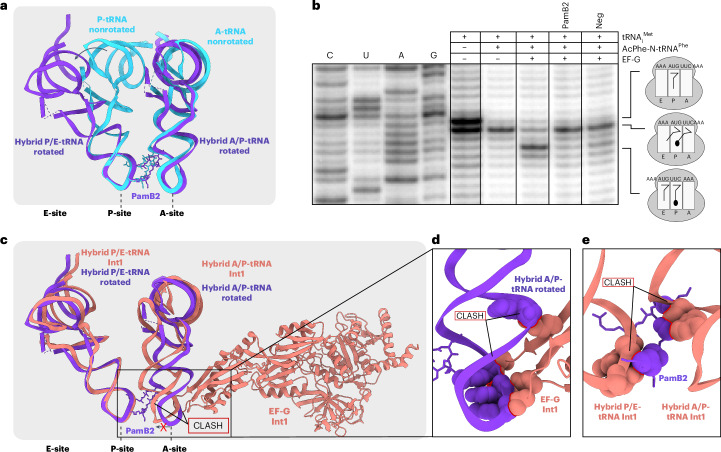
PamB2 inhibits tRNA_2_–mRNA translocation. **a**, Superimposition of PamB2 and tRNAs of the nonrotated (light blue) and rotated (purple) PamB2–70S complexes. **b**, Toeprinting assay monitoring the effect of PamB2 on EF-G dependent translocation, with initiator tRNA^fMet^ and *N*-AcPhe-tRNA^Phe^ in the absence of drugs and in the presence of the translocation inhibitor negamycin^22^. Toeprinting assays were performed in duplicate, with the duplicate gel shown in the source data. **c**, Superimposition of PamB2 and hybrid tRNAs of the rotated (purple) PamB2–70S complex with hybrid tRNAs and EF-G bound to the *E. coli* 70S ribosome in the Int1 state (salmon, PDB ID 7N2V)^19^. **d**,**e**, Sphere representation of the hybrid A/P-tRNA anticodon-stem loop of the rotated PamB2 complex sterically clashing with EF-G (salmon, PDB ID 7N2V)^19^ (**d**) and of the hybrid A/P- and P/E-tRNAs of the Int1 state (PDB ID 7N2V)^19^ (**e**) clashing with PamB2 (purple). Steric clashes are highlighted with red lines. Source data

**Extended Data Fig. 6:**
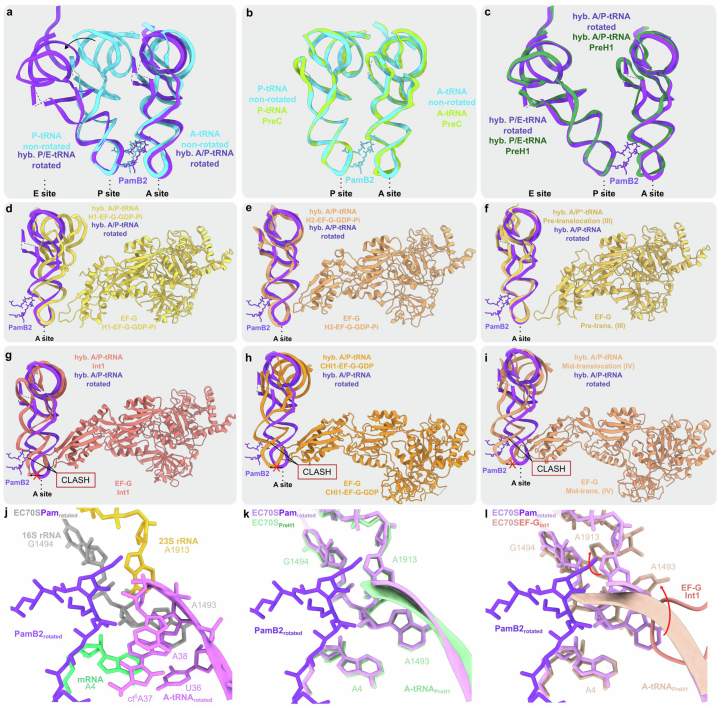
PamB2 inhibits tRNA_2_-mRNA translocation. **a**, Superimposition of the P- and A-site tRNA of the non-rotated PamB2 complex (light blue) and the hybrid A/P- and P/E-tRNA of the rotated PamB2 complex (dark purple). **b**, Superimposition of the P- and A-tRNA of the non-rotated PamB2-complex (light blue) and the P- and A-tRNA of the PreC state (light green, PDB ID 7N1P)^19^. **c**, Superimposition of the hybrid A/P- and P/E-site tRNA of the rotated PamB2-complex (dark purple) and the hybrid A/P- and P/E-site tRNA of the PreH1 state (dark green, PDB ID 7N2U)^19^. **d–i**, Superimposition of the hybrid A/P-site tRNA of the rotated PamB2-complex (dark purple) and the hybrid A/P-site tRNA and EF-G of the (**d**) H1-EF-G-GDP-Pi state (light yellow, PDB ID 7PJV)^21^, (**e**) H2-EF-G-GDP-Pi state (light orange, PDB ID 7PJW)^21^ and (**f**) pre-translocation (III) state (yellow, PDB ID 7SSL)^20^, (**g**) Int1 state (salmon, PDB ID 7N2V)^19^, (**h**) CHI1-EF-G-GDP state (orange, PDB ID 7PJY)^21^, (**i**) mid-translocation (IV) state (light orange, PDB ID 7SSD)^20^. **j–l**, PamB2 of the rotated complex (**j**) with surrounding hybrid A/P-tRNA (purple), 16S rRNA nucleotides (grey), 23S rRNA nucleotides (yellow) and the A-site codon of the mRNA (cyan) superimposed with (**k**) with the 70S *E. coli* ribosome in the PreH1 state (light green, PDB ID 7N2U)^19^ and (**l**) with the 70S *E. coli* ribosome and EF-G of the Int1 state shown as ribbon (salmon, PDB ID 7N2V)^19^.

Careful examination of the tRNAs in the PamB2-bound elongation complexes revealed the presence of additional density attached to the CCA-end of the A-site tRNA in the nonrotated elongation state and to the A/P-tRNA in the rotated hybrid state, indicating that peptide bond formation has already occurred in these complexes (Supplementary Fig. 3a,b). This suggests that PamB2 does not interfere with the decoding and accommodation by the A-tRNA, nor peptide bond formation, and also allows the ribosome to oscillate between the canonical and hybrid pretranslocation states (Fig. 3a). During normal translation, elongation factor EF-G binds and translocates the tRNA_2_–mRNA complex into the P- and E-sites, forming a posttranslocational state of the ribosome^19–21^. The accumulation of pretranslocational states in the presence of PamB2, as well as the absence of posttranslocation states (Fig. 1b–e and Extended Data Fig. 6a–c), suggests that PamB2 may interfere with the process of translocation. To directly assess this, we analyzed the effect of PamB2 on EF-G-dependent translocation using the toeprinting assay. Ribosome complexes were formed with tRNA^fMet^ in the P-site and *N*-AcPhe-tRNA^Phe^ in the A-site, with toeprinting revealing a band corresponding to the expected pretranslocation state in the absence of EF-G (Fig. 3b). On addition of EF-G, but in the absence of PamB2, the toeprinting band shifted by three nucleotides, indicating that the A- and P-site tRNAs were translocated to the P- and E-sites (Fig. 3b). In contrast, little to no shift in the toeprint band was observed when the same reactions were performed in the presence of PamB2 or the control antibiotic negamycin, which was reported previously to interfere with translocation^22^. Hence, we conclude that binding of PamB2 to ribosome inhibits the process of translocation (Fig. 3b). Comparison with recent structures of EF-G-bound translocation intermediates provides a structural rationale for the PamB2-mediated translocation inhibition^19–21^. While the initial binding of EF-G to the ribosome may be possible in the presence of PamB2 (Extended Data Fig. 6d–f)^20,21^, the subsequent steps where EF-G accommodates, releases the decoding center^23^ and promotes a shift of the anticodon stem of the A/P-site tRNA that would lead to severe clashes with PamB2 (Fig. 3c–e and Extended Data Fig. 6g–i)^19–21^. Moreover, in the early translocation intermediate with EF-G, A1913 rotates away from its position in the hybrid states^19–21^, which would require disruption of interactions between A1913 and PamB2 (Extended Data Fig. 6j–l). Collectively, this leads us to suggest that the interactions of PamB2 with the anticodon-stem loop of the A-site tRNA, as well as with the extended conformation of A1913, would interfere with productive translocation and thereby inhibit protein synthesis.

### Influence of A-site mRNA context on PamB2 inhibition

While the translocation assay (Fig. 3b) and structures of PamB2 bound to pretranslocation complexes (Fig. 1b–e) support the conclusion that PamB2 interferes with the EF-G mediated translocation process, our initial toeprinting assay indicated that it was not the first translocation step that was inhibited, but rather the second (Extended Data Fig. 2). If the first translocation reaction was inhibited, then ribosomes would be trapped with the AUG start codon in the P-site being decoded by the initiator tRNA^fMet^ and with a UUG codon in the A-site. While the density indicates that the initiator tRNA^fMet^ is present in the P-site of the P-tRNA-only volume (Supplementary Fig. 4a), the density for the mRNA codons and tRNAs in the A- and P-sites in structures of the PamB2-bound pretranslocation states indicated that one round of translocation had occurred before stalling of the complex (Supplementary Fig. 4b–e), that is UUG and AUA codons are in the P- and A-sites being decoded by tRNA^Leu^ (anticodon 5′-_35_CAA_37_-3′) and tRNA^Ile^ (anticodon 5′-_35_CAU_37_-3′), respectively (Supplementary Fig. 4d,e). Moreover, we observe extra density for the 2-methylthio-N6-isopentenyladenine (ms^2^i^6^A) at position 37 of the P-site tRNA^Leu^ as well as the cyclic N6-threonylcarbamoyladenosine (ct^6^A) at position 37 of the A-site tRNA^Ile^ (Supplementary Fig. 4f–i). Collectively, these findings suggest that PamB2 allowed the first translocation on the MLIFstop-mRNA, but prevented the second translocation reaction from taking place.

**Fig. 4:**
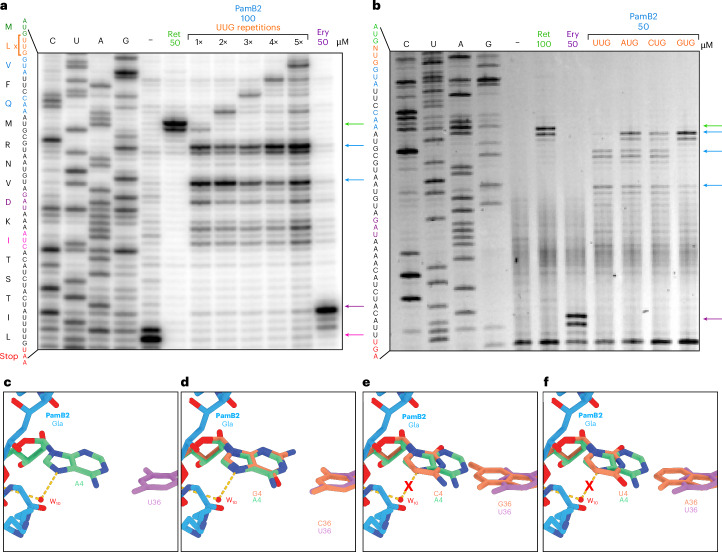
Influence of A-site mRNA context on PamB2 inhibition. **a**,**b**, Toeprinting assays monitoring the position of ribosomes on the wildtype ErmBL mRNA in the presence of water, 50 or 100 µM retapamulin (Ret), 50 µM erythromycin (Ery) and an ErmBL mRNA with an increasing number of UUG repetitions in the presence of 100 µM PamB2 (**a**) and with an ErmBL mRNA with the second codon mutated to UUG, AUG, CUG, GUG (orange) in the presence of 50 µM PamB2 (**b**). Arrows indicate the stalling sites on the isoleucine catch codon in the presence of mupirocin (pink), at initiation (green), on the erythromycin-ErmBL stalling site (purple) and stalling induced by PamB2 (blue). Toeprinting assays were performed in duplicate, with the duplicate gel shown in the Source Data. **c**–**f**, Water (red) mediated interaction (dashed line) of PamB2 (blue) and the first nucleotide of the mRNA of the A-site codon (cyan), and superimposed with in silico mutated first position of the A-site codon (orange) to guanine (**d**), cytosine (**e**) or uracil (**f**). The loss of the water-mediated interaction is indicated by a red cross. Source data

To investigate whether it is the initiation context that interferes with the action of PamB2 or whether PamB2 acts in a sequence context-specific manner, we generated a series of model mRNA templates encoding a short ErmBL protein^24,25^ with 1–5 repeats of the UUG codon directly following the AUG start codon (Fig. 4a). In the absence of antibiotic, ribosomes initiate on the AUG start codon of the wildtype ErmBL mRNA (with a single UUG codon), and translate uninterrupted to the twelfth codon (AUC encoding Ile), where they become trapped due to the presence of the Ile-tRNA synthetase inhibitor mupirocin that was added to all reactions (Fig. 4a). Addition to the control reaction of the antibiotic retapamulin traps ribosomes on the AUG start codon^26^, whereas the macrolide erythromycin leads to the accumulation of ribosomes stalled with the tenth CAU codon (encoding Asp) in the P-site (Fig. 4a), as we observed previously on the ErmBL mRNA^24,25^. Unlike retapamulin, the presence of PamB2 did not lead to a strong accumulation of ribosomes on the AUG start codon of the wildtype (1×UUG) ErmBL mRNA template, but rather ribosomes became stalled only once the second *ermBL* codon (UUG) moved into the P-site (Fig. 4a), as we observed for the MLIFstop-mRNA (Extended Data Fig. 2). While the insertion of UUG codons into the ErmBL mRNA shifted the band for initiating ribosomes upward in the gel as expected, the first main stalling bands remained constant (Fig. 4a), indicating that in the presence of PamB2, ribosomes can translate through stretches of up to five UUG codons unhindered. We conclude therefore that the lack of effect of PamB2 on the first translocation event in the wildtype ErmBL mRNA is not related to the initiation context, but rather the presence of the UUG codon in the A-site. We also note that unlike for the short MLIFstop-mRNA, additional bands were observed on the longer ErmBL mRNA indicating that a subset of ribosomes also become stalled at subsequent sites in the mRNA, for example, with the fourth UUC (encoding Phe) in the P-site, but not the third GUA codon in the P-site (Fig. 4a).

An initial examination of the nonstalling contexts revealed that that the presence of U in the first position of the A-site codon antagonizes PamB2 action. Therefore, to test whether the nature of the A-site codon can influence the efficiency of PamB2-mediated translocation inhibition, we mutated the U in the first position of the A-site codon of the ErmBL mRNA to A, C and G (Fig. 4b). In contrast to U in the first position where little to no inhibition of the first translocation event was observed (Fig. 4b), clear toeprint bands were observed with each of the other nucleotides, indicating that ribosomes accumulate with the AUG start codon in the P-site when the A-site codon was changed from UUG to AUG, CUG or GUG (Fig. 4b). Although the inhibition by PamB2 with C in the first position appeared to be stronger than with U, it was reproducibly weaker than with A and G (Fig. 4b). The inhibition with G in the first position of the A-site codon appeared to be the strongest (Fig. 4b). Although PamB2 does not directly interact with the first position of the A-site codon, we observed that a water-mediated interaction is present between the backbone amide of mDap2 via water W_10_ with the N7 of A in the first position of the A-site codon in the ribosome stalled on the MLIFstop-mRNA (Fig. 4c). A similar interaction would be maintained with a G in the first position (Fig. 4d) where we observe strong inhibition (Fig. 4b), but would not be possible with C or U (Fig. 4e,f) where inhibition was weaker (Fig. 4b).

### Influence of A37 modification of A-tRNA on PamB2 inhibition

**Fig. 5:**
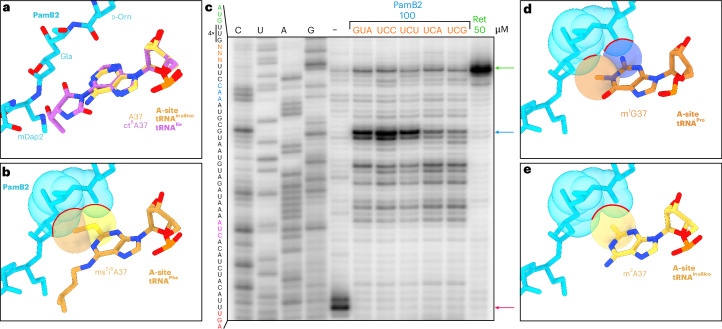
Influence of A37 modification of A-tRNA on PamB2 inhibition. **a**, PamB2 (light blue) and the modified A-site tRNA residue cyclic N6-threonylcarbamoyladenine (ct6) in position 37 (purple) from the nonrotated PamB2 complex superimposed with an in silico model of an unmodified A37 (yellow). **b**, Superimposition of PamB2 from **a** with the 2-methylthio-N6-isopentenyladenine (ms^2^i^6^, light orange) at position 37 of the A-site tRNA^Phe^ from the *T. thermophilus* 70S ribosome preattack state (PDB ID 1VY5)^27^ shown as sphere representation with clashes indicated by red lines. **c**, Toeprinting assay monitoring the position of ribosomes on the (UUG)_4_-ErmBL mRNA in the presence of water (–), 50 µM retapamulin (Ret, green) and 100 µM PamB2 (light blue). The seventh codon was modified to different serine codons (orange). Arrows indicate stalling for the isoleucine catch codon in the presence of mupirocin (pink), the initiation (green) and PamB2-induced stalling (light blue). Toeprinting assays were performed in duplicate, with the duplicate gel present in the Source Data. **d**,**e**, Superimposition of PamB2 from **a** with 1-methyl-guanine (m^1^G, dark orange) at position 37 of the A-site tRNA^Pro^ on the *T. thermophilus* 70S ribosome (PDB ID 6NUO)^41^ (**d**) and an in silico modified 2-methyl-adenine (m^2^A, yellow) shown as sphere representation with steric clashes indicated by red lines (**e**). Source data

While the water-mediated interaction between PamB2 and the first position of the A-site codon may contribute to the specificity of stalling of PamB2, it does not rationalize the difference in efficiency of inhibition of PamB2 that we observed between U and C in the first codon position (Fig. 4b). Therefore, we considered whether the nature of the tRNA in the A-site may also contribute, especially given that we noticed interactions between PamB2 and nucleotides A37 and A38 of the A-site tRNA (Fig. 2e,f). Since we observe no inhibition by PamB2 when Phe-tRNA decodes UUC, we superimposed a ribosome structure with Phe-tRNA in the A-site^27^ and immediately noticed that tRNA^Phe^ bears a 2-methylthio-N6-isopentenyladenine (ms^2^i^6^A) at position 37, with the 2-methylthio moiety encroaching on the PamB2 binding site (Fig. 5a,b). In fact, with one exception (see later), all tRNAs that decode mRNA codons beginning with U have ms^2^i^6^A37, which is proposed to help stabilize the weaker U–C codon–anticodon interaction between the mRNA and the tRNA^28^. Consistently, the ribosome is not inhibited by PamB2 when tRNA^Leu^ (with ms^2^i^6^A37) decodes UUG in the A-site (Fig. 4a), and similar results would be expected for tRNA^Ser^ decoding UCU/UCA/UCG, tRNA^Tyr^ decoding UAU/UAC, tRNA^Cys^ decoding UGU/UGC and tRNA^Trp^ decoding UGG. The one exception is tRNA^Ser^ that decodes UCU and UCC but has A37 unmodified^28^. To directly test this, we generated a series of mRNA templates based on the ErmBL-(UUG)_4_ mRNA where we changed the seventh GUA (Val) codon to each of the four serine codons UCC, UCU, UCA and UCG and performed the toeprinting assay in the presence of PamB2 (Fig. 5c). As hypothesized, strong stalling was observed at the UCC and UCU codons, which are decoded by the tRNA^Ser^ isoacceptor lacking any modification at position A37, whereas only weak stalling was observed at the UCA and UCG codons, which are decoded by the tRNA^Ser^ isoacceptor bearing ms^2^i^6^A37 (Fig. 5c). Thus, we conclude that PamB2 is a poor inhibitor of translocation when it has to compete with the A-site tRNA containing ms^2^i^6^A37.

Although PamB2 inhibited translation when tRNA^Leu^ decoded CUG in the A-site, the extent of inhibition was relatively weak (Fig. 4a). Therefore, we superimposed a ribosome structure with tRNA^Leu^ in the A-site^17^ and recognized that the m^1^G at position 37 of tRNA^Leu^ would clash with PamB2 due to a steric hindrance between the N2 group of m^1^G37 of the A-tRNA and the d-Orn of PamB2 (Fig. 5d). In fact, most tRNAs decoding CNN codons, including CUN by tRNA^Leu^, CCN by tRNA^Pro^ as well as CGG by tRNA^Arg^ contain m^1^G37 (Supplementary Fig. 5)^28^. The exceptions are tRNA^His^ and tRNA^Gln^ that decode CAU/C and CAA/G, as well as tRNA^Arg^ that decodes CGU/C/A, however, all of these tRNAs have m^2^A37 (ref. ^28^) (Supplementary Fig. 5) that would also be predicted to clash with the d-Orn of PamB2, similar to m^1^G37 (Fig. 5d,e and Supplementary Fig. 5). Collectively, we conclude that the efficiency of translocation inhibition by PamB2 is directly influenced by the nature of the A-site tRNA and in particular by modifications at position A37, such as m^1^G37, possibly m^2^A37 and especially ms^2^i^6^A37 (Supplementary Fig. 5) where the steric overlap with the drug is largest.

## Discussion

**Fig. 6:**
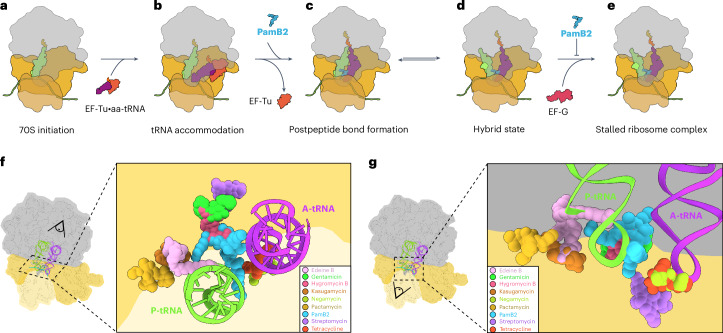
Mechanism of action of PamB2 and relative binding site of PamB2 compared to other antibiotics. **a–e**, Model for the mechanism of action of PamB2 during translation. **a**,**b**, PamB2 does not bind stably to the initiation state with P-site tRNA only (**a**), nor during delivery and decoding of the aminoacyl-tRNA to the A-site by EF-Tu (**b**). **c**,**d**, PamB2 binds stably to pretranslocation states with A- and P-site tRNAs in nonrotated state and does not prevent peptide bond formation (**c**), as well as the rotated hybrid state with A/P- and P/E-tRNAs (**d**). **e**, Stable binding of EF-G is prevented by PamB2 thereby preventing translocation and trapping the ribosome in the pretranslocational states. **f**,**g**, View from the 50S (**f**) and 30S (**g**) subunit of PamB2 (light blue) superimposed with edeine B (pink, PDB ID 1I95)^42^, gentamicin (neon green, PDB ID 8CGU)^16^, hygromycin B (hot pink, PDB ID 8CAI)^16^, kasugamycin (dark orange, PDB ID 8CEP)^16^, negamycin (light green, PDB ID 4W2I)^22^, pactamycin (yellow, PDB ID 4W2H)^43^, streptomycin (pink, PDB ID 8CAI)^16^ and tetracycline (pink, PDB ID 8CF1)^16^, shown in sphere representation on the 30S subunit (head, light yellow; body, yellow), the 50S subunit (gray) and P- (green) and A-tRNA (purple).

Based on our results, we propose a model for the mechanism of action of how PamB2 binds to the ribosome and inhibits protein synthesis (Fig. 6a–e). Our data suggest that PamB2 does not interfere with translation initiation (Fig. 6a), nor the initial EF-Tu-mediated decoding steps during translation elongation (Fig. 6a,b), but rather binds stably to the ribosome once the A-site tRNA becomes accommodated on the large ribosomal subunit (Fig. 6c). Our structural data indicate that PamB2 does not prevent peptide bond formation (Fig. 6c), nor the ribosome from adopting the rotated conformation with hybrid state A/P- and P/E-site tRNAs (Fig. 6d). Instead, we demonstrate that PamB2 interferes with the subsequent translocation step, where the tRNA_2_–mRNA complex is moved through the ribosome to occupy the P- and E-sites with the help of EF-G (Fig. 6e). We suggest that translocation is inhibited because PamB2 traps a pretranslocational state that is incompatible with stable binding of elongation factor EF-G (Figs. 3c–e and 6d,e). Based on the high similarity in chemical structures (Extended Data Fig. 1), we propose that the mechanism of action described here for PamB2 will be similar, if not identical, for other paenilamicin congeners (PamB1, PamA1 and PamA2)^3^ as well as the related compound galantin I (ref. ^6^). Notably, modifications at the N-terminal amine of Agm/Cad in each of these congeners with either an *N*-acyl-d-Asn^8^ or an acetyl moiety^7^ will interfere with ribosome binding (Extended Data Fig. 4a–d), which thus rationalizes the self-resistance strategies of the producer *P. larvae*.

One of the most unexpected findings of our study was that pretranslocation translation complexes formed at distinct mRNA sites were refractory to the action of PamB2. Thus, PamB2 does not inhibit each and every round of translation elongation indiscriminately, but rather can be considered as a context-specific translocation inhibitor. The best understood context-specific translation inhibitors are those that target the large subunit, where their inhibitory action is influenced by the sequence of the nascent polypeptide chain being synthesized^29^, for example, macrolides and ketolides^30–32^, oxazolidinones and phenicols^33–35^ and more recently orthosomycins and tetracenomycins^36–38^. However, there are other examples of context-specific antibiotics that target the small subunit where their inhibitory activity appears to also be influenced by the nature of the mRNA and/or tRNA, such as pactamycin^39^, negamycin^22^ and kasugamycin^40^, yet a structural basis for their specificity has so far been lacking. By contrast, we provide a structural basis for the context-specificity of PamB2, demonstrating that translation is less affected by the action of PamB2 when the A-site is occupied by tRNAs bearing modifications of nucleotide A37. This is exemplified by most tRNAs decoding UNN codons that bear a ms^2^i^6^A37 modification, such that the 2-methylthio moiety would be predicted to sterically clash with the binding position of PamB2 on the ribosome (Fig. 5a,b). A weaker refractory action was also observed with the A-site tRNAs that decode CNN codons due to the presence of either m^1^G37 or m^2^A37, which would also lead to clashes with PamB2 (Fig. 5d,e). In *E. coli*, no other tRNAs have modifications at the A37 position that would be predicted to interfere with PamB2 activity^28^, however, we cannot exclude that it is different in other bacteria. Despite this context-specific action, PamB2 is a potent inhibitor of protein synthesis, with an IC_50_ of 0.4 μM. This is most likely because the translation of most, if not all, mRNAs involves translation of many codons that are read by tRNAs lacking modifications on the C2 position of A37 and are therefore susceptible to the action of PamB2.

Despite the suggested similarity of paenilamicins to edeine^4^, we show here that the PamB2 binding site is principally different from that reported for edeine (Fig. 6f,g). In fact, the binding site of PamB2 identified here is distinct from that reported for any other class of translation inhibitor (Fig. 6f,g). The only antibiotic with a binding site that slightly overlaps with PamB2 is the aminoglycoside hygromycin B (Fig. 6f,g), which binds within helix 44 and interacts with a putative K^+^ ion^16^ that is also coordinated by PamB2 (Fig. 2d). However, most of the rest of the PamB2 interactions with the ribosome are distinct from those of hygromycin B. The unique binding site and interactions of PamB2 with the ribosome compared to other clinically used compounds suggest that there is little chance for cross-resistance with PamB2. Together with the good activity against methicillin-resistant *S. aureus*^4^, this makes paenilamicins an attractive class of compounds for the future development of antimicrobial agents to combat drug-resistant pathogenic bacteria.

## Methods

### Synthesis of paenilamicins

Synthetic PamB2 and its *N*-acetylated form were produced as previously reported^4,7^. Briefly, PamB2 was synthesized by synthesizing and combining the left-hand side Agm-containing with the right-hand side Spd-containing fragments^4,7^, whereas the *N*-acetylated form of PamB2 was acquired by incubation of PamB2 with PamZ^4,7^.

### Toeprinting assays

Toeprinting reactions for Fig. 4b and Extended Data Fig. 2 were performed as described previously^14^. Briefly, reactions were performed with 6 μl of PURExpress in vitro protein synthesis system (New England Biolabs). The reactions were carried out with different primers and templates (Supplementary Tables 2 and 3). The reactions contained 340 ng of the respective mRNA template and were supplemented with the different compounds as specified. The translation reactions were incubated for 30 min at 37 °C. The reverse transcription reaction was carried out using AMV RT and primer NV*1-Alexa 647 (5′-GGTTATAATGAATTTTGCTTATTAAC-3′). The translation reactions were incubated with the reverse transcriptase and the primer for 20 min at 37 °C. mRNA degradation was carried out by the addition of 1 µl of 5 M NaOH. The reactions were neutralized with 0.7 µl of 25% HCl, and nucleotide removal was performed with the QIAquick Nucleotide Removal Kit (Qiagen). The samples were dried under vacuum for 2 h at 60 °C for subsequent gel electrophoresis. The 6% acrylamide gels were scanned on a Typhoon scanner (GE Healthcare).

Toeprinting reactions for Figs. 4a and 5c were performed as described previously^44^. Briefly, reactions were performed with 5 μl of PURExpress in vitro protein synthesis system. The reactions contained 0.1 pmol of the respective DNA template (Supplementary Table 2) and were supplemented with retapamulin, erythromycin, mupirocin or PamB2 as specified. The transcription–translation reactions were incubated 20 min at 37 °C. Subsequently, reverse transcription was performed for 10 min at 37 °C using AMV RT and the radiolabeled NV*1-primer. Reactions were stopped by the addition of 1 µl of 10 M NaOH and then neutralized with 0.8 µl of concentrated HCl. Subsequently, 200 µl of the stop buffer (0.3 M sodium acetate (pH 5.5), 5 mM EDTA and 0.5% SDS) was added and phenol extraction was performed. The obtained complementary DNA was precipitated in ethanol for subsequent gel electrophoresis.

### Translocation assay

Translocation assays were performed as previously described^22^ using the MFKAFK template^45^ (Supplementary Table 2). Reactions were prepared by incubating tight-coupled ribosomes (0.7 µM) with mRNA (0.5 µM) and tRNA_i_^Met^ (1 µM) for 20 min at 37 °C in the Pure System Buffer (5 mM potassium phosphate (pH 7.3), 9 mM Mg(OAc)_2_, 95 mM potassium glutamate, 5 mM NH_4_Cl, 0.5 mM CaCl_2_, 1 mM spermidine, 8 mM putrescine and 1 mM dithiothreitol) and for additional 10 min at 37 °C with 2 μM of *N*-acetyl-Phe-tRNA^Phe^. At the time of *N*-acetyl-Phe-tRNA^Phe^ addition, the reactions were supplemented with PamB2 or negamycin as specified. The translocation reaction was initiated by addition of 1 µl of EF-G–GTP mixture (1.2 µM and 3.2 mM). After 5 min of incubation at 30 °C, 2 µl of reverse transcriptase and/or dNTPs mixture was added. The reactions were stopped after another 5 min at 30 °C by addition of 200 µl of the stop buffer and subsequent phenol extraction. The obtained complementary DNA was precipitated in ethanol for subsequent gel electrophoresis.

### Preparation of complexes for structural analysis

PamB2-ribosome complexes were generated by in vitro transcription–translation reactions in PURExpress in vitro protein synthesis system (New England Biolabs) with the same reaction mix as described earlier in the toeprinting assays. Complex formation reactions were carried out on MLIFstop toeprint DNA template in a 48 µl of reaction in presence of 50 µM PamB2. The reaction was incubated for 15 min at 37 °C. The reaction volume was then split: 42 µl were used for complex generation and 6 µl were used for toeprinting analysis. Ribosome complexes were isolated by centrifugation in 900 µl of sucrose gradient buffer (containing 40% sucrose, 50 mM HEPES-KOH, pH 7.4, 100 mM KOAc, 25 mM Mg(OAc)_2_ and 6 mM 2-mercaptoethanol) for 3 h at 4 °C with 80,000*g* in a Optima Max-XP Tabletop Ultracentrifuge with a TLA 120.2 rotor. The pelleted complex was resuspended in Hico buffer (50 mM HEPES-KOH, pH 7.4, 100 mM KOAc, 25 mM Mg(OAc)_2_) supplemented with 50 µM PamB2, then incubated for 15 min at 37 °C.

### Preparation of cryo-EM grids and data collection

Sample volumes of 3.5 μl (eight at an optical density of 260 nm per milliliter) were applied to grids (Quantifoil, Cu, 300 mesh, R3/3 with 3-nm carbon) that had been freshly glow-discharged using a GloQube (Quorum Technologies) in negative charge mode at 25 mA for 90 s. Sample vitrification was performed using an ethane-propane mixture (37:63) in a Vitrobot Mark IV (Thermo Fisher Scientific), the chamber was set to 4 °C and 100% relative humidity and blotting was done for 3 s with no drain or wait time. Frozen cryo-EM grids were imaged on a TFS 300 kV Titan Krios at the Dubochet Center for Imaging EPFL (Lausanne, Switzerland). Images were collected on Falcon IV direct detection camera in counting mode using the EPU and AFIS data collection scheme with a magnification of ×96,000 and a total dose of 60 electrons per square angstrom (e^−^/Å^2^) for each exposure, and defocus ranging from −0.4 to −0.9 μm. In total, 7,638 videos were produced in EER format at a pixel size of 0.8 Å per pixel.

### Single-particle reconstruction of PamB2-stalled ribosome complexes

RELION v.4.0.1 (ref. ^46^) was used for processing, unless otherwise specified. For motion correction, RELION’s implementation of MotionCor2 with 4×4 patches and for initial contrast transfer function (CTF) estimation CTFFIND v.4.1.14 (ref. ^47^) was used. From 7,638 micrographs, 611,189 particles were picked using crYOLO v.1.8.04b47 with a general model^48^. Then 562,816 ribosome-like particles were selected after two-dimensional classification and extracted at three times decimated pixel size (2.4 Å per pixel) (Supplementary Fig. 1b,c). An initial three-dimensional (3D) refinement was done using a *E. coli* 70S reference map (EMD-12573)^30^. Particles were 3D classified for 100 iterations and resulted in four classes of which a nonrotated 70S class with A-, P- and E-site tRNAs (65.0%, 365,773 particles) and a rotated 70S with hybrid A/P- and P/E-tRNA (22.4%, 126,259 particles) (Supplementary Fig. 1d) were further subsorted. Subsorting was done for 100 iterations for both classes individually (Supplementary Fig. 1e,g), yielding two classes of nonrotated 70S with A-, P- and substoichiometric E-site tRNA (52.5%, 295,568 particles) and rotated 70S class with hybrid A/P- and P/E-tRNA (16.7%, 93,773 particles), respectively. Focus-sorting was performed with partial particle subtraction using a mask surrounding the tRNAs for the particles containing nonrotated 70S with A-, P- and substoichiometric E-site tRNA and 3D classified for 100 iterations yielding six classes. Classes containing A-, P- and E-site tRNA (31.4%, 176,827 particles), as well as classes containing just P-site tRNA (15.1%, 84,771 particles) were combined and further processed (Supplementary Fig. 1f). All resulting classes were 3D refined (with a solvent mask), CTF refined (fourth-order aberration, anisotropic magnification and per-particle defocus value estimation), Bayesian polished, again CTF refined and after a final 3D refinement yielded a final average resolution of 2.2 Å (at FSC_0.143_) for the postprocessed masked reconstruction of the nonrotated 70S complex containing A-, P- and sub-E-site tRNA (Supplementary Fig. 1h), a final average resolution of 2.4 Å (at FSC_0.143_) for the postprocessed masked reconstruction of the 70S complex containing P-tRNA (Supplementary Fig. 1i) and a final average resolution of 2.3 Å (at FSC_0.143_) for the postprocessed masked reconstruction of the rotated 70S complex containing hybrid A/P-, and P/E-tRNAs (Supplementary Fig. 1f). To estimate local resolution values Bsoft v.2.1.1 (ref. ^49^) was used on the half-maps of the final reconstructions (blocres -sampling 0.8 -maxres -boc 20 -cutoff 0.143 -verbose 1 -origin 0,0,0 -Mask half_map1 half_map 2) (Extended Data Fig. 3d–m).

### Molecular modeling of the PamB2-ribosome complexes

The molecular models of the 30S and 50S ribosomal subunits were based on the *E. coli* 70S ribosome (Protein Data Bank (PDB) ID 7K00)^50^. PamB2 and in silico modified versions of paenilamicins were generated and restraints created using aceDRG^51^ and modeled de novo. The nonrotated and rotated 70S complexes were assembled with tRNA^Leu^ and tRNA^Ile^ used from the drosocin-stalled 70S complexes (PDB ID 8AM9)^12^. The initiation complex was assembled with an initiator fMet-tRNA (PDB ID 1VY4)^27^ in the P-site. Modifications of rRNA nucleotides and tRNA^Leu^ and tRNA^Ile^ were generated using aceDRG^51^. Starting models were rigid body fitted using ChimeraX v.1.6.1 (ref. ^52^) and modeled using Coot v.0.9.8.92 (ref. ^53^) from the CCP4 software suite v.8.0.017 (ref. ^54^). The sequence for the tRNAs were adjusted based the appropriate anticodons corresponding to the mRNA. Final refinements were done in REFMAC 5 (ref. ^55^) using Servalcat v.0.4.28 (ref. ^56^). The molecular models were validated using Phenix comprehensive cryo-EM validation in Phenix v.1.20.1-4487 (ref. ^57^).

### Eukaryotic in vitro translation assays

The effect of PamB2 on eukaryotic translation was determined using the Rabbit Reticulocyte Lysate System (Promega) as previously described^58^. Briefly, 6-μl reactions, with or without PamB2 were mixed according to the manufacturer’s description and incubated for 30 min at 32 °C with shaking (600 rpm). Each reaction was stopped by adding 3 μl of cycloheximide (600 μM). All samples were diluted with 40 μl of Luciferase assays substrate (Promega) into a white 96-well chimney flat-bottom microtiter plate (Greiner). The luminescence was then measured using a Tecan Infinite M1000 plate reader. Relative values were determined by defining the luminescence value of the sample without inhibitor as 100%.

### In vitro cytotoxicity assay

NIH/3T3 murine fibroblasts (ATCC CRL-1658) were grown to subconfluence (~80%) at 37 °C in the presence of 5% CO_2_ in a T-25 flask (Sarstedt) containing 6 ml of Dulbecco’s modified Eagle’s medium (DMEM, Sigma-Aldrich) supplemented with 100 U ml^−1^ penicillin (Sigma), 100 μg ml^−1^ streptomycin (Sigma), 2 mM l-glutamine and 10% (v/v) fetal bovine serum (EuroClone). Cells were removed from the flask by 5-min incubation at 37 °C with 2 ml of trypsin/EDTA (500 mg l^−1^ trypsin, 371 mg l^−1^ EDTA, EuroClone SpA) that was subsequently neutralized with 2 ml of DMEM. Detached cells were counted in a Bürker chamber in the presence of 1 mg ml^−1^ trypan-blue to visually check the absence of nonviable cells and finally diluted in medium to the desired working concentration of 5 × 10^4^ cells per milliliter. Then, 100 µl of this suspension was aliquoted in the wells of a 96-well flat-bottom microtiter tissue culture plate (Sarstedt) to seed 5,000 cells per well. The plate was incubated for 18 h at 37 °C and 5% CO_2_ to allow cell adhesion and proliferation. The next day, the exhausted medium was removed from each well and replaced with 100 μl of new DMEM medium containing the desired concentrations of PamB2 or cycloheximide, while controls for these compounds were received the solvents of these compounds, namely, water or dimethylsulfoxide, respectively. The plate was further incubated 48 h at 37 °C and 5% CO_2_. Then the cytotoxicity of the PamB2 and cycloheximide was assessed by the 3-(4,5-dimethylthiazol-2-yl)-2,5-diphenyltetrazolium bromide (MTT) assay. The exhausted medium containing the compounds was disposed from each well of the plate and substituted with 125 μl of DMEM containing 1 mg ml^−1^ MTT (Merck Life Science S.r.l.). The plate was then incubated for 4 h at 37 °C and 5% CO_2_ in the dark. Subsequently, the MTT solution was carefully removed from each well to prevent loss of MTT crystals. To resuspend the MTT crystals, 100 μl of 10% (w/v) IGEPAL in 10 mM HCl (Merk Life Science S.r.l.) were added to each well and the plate was incubated overnight at 37 °C and 5% CO_2_. The next day, absorbance was measured at 570 nm using a Nanoquant Infinite-M200Pro plate reader (Tecan). Viability was calculated by assigning the absorbance of the untreated controls that received water only as 100%. Results are the average calculated on three independent experiments performed as internal triplicates (*n* = 9).

### Figures

UCSF ChimeraX v.1.6.1 (ref. ^52^) was used to isolate density, align molecular models and visualize density images and structural superpositions. Figures were assembled with Inkscape v.1.3 (latest development release, regularly updated).

### Reporting summary

Further information on research design is available in the Nature Portfolio Reporting Summary linked to this article.

## Online content

Any methods, additional references, Nature Portfolio reporting summaries, source data, extended data, supplementary information, acknowledgements, peer review information; details of author contributions and competing interests; and statements of data and code availability are available at 10.1038/s41589-024-01752-9.

## Supplementary information

Supplementary Information Supplementary Figs. 1–5, Tables 1–3 and References.

Reporting Summary

Supplementary Video 1 Cryo-EM map of the PamB2–70S revealing PamB2 binding site between A- and P-site tRNAs with direct and water-mediated interactions with mRNA (green), P-tRNA (lime), A-tRNA (purple), 16S rRNA (gray) and 23S rRNA (yellow).

## Source data

Source Data Fig. 3 Unprocessed gel for Fig. 3a and duplicate gel.

Source Data Fig. 4 Unprocessed gel for Fig. 4a,b and duplicate gels.

Source Data Fig. 5 Unprocessed gel for Fig. 5c and duplicate gel.

Source Data Extended Data Fig. 2 Unprocessed gel for Extended Data Fig. 2 and duplicate gel.

Source Data Extended Data Fig. 5 Raw data in triplicate for graph in Extended Data Fig. 5b,c.

## Data Availability

Micrographs have been deposited as uncorrected frames in the Electron Microscopy Public Image Archive with the accession codes EMPIAR-12080. Cryo-EM maps have been deposited in the Electron Microscopy Data Bank with accession codes EMD-18950 (Nonrotated 70S PamB2 complex), EMD-19004 (Rotated 70S PamB2 complex) and EMD-50296 (Initiation 70S complex). Molecular models have been deposited in the PDB with accession codes 8R6C (Nonrotated 70S PamB2 complex), 8R8M (Rotated 70S PamB2 complex) and 9FBV (Initiation 70S complex). Structures from previous studies were used in this work for comparison, alignments and for modeling and are available in the PDB under the IDs 1I95, 1VY4, 1VY5, 4V6Z, 4V8D, 4W2I, 4W2H, 6NUO, 6WD0, 6WD2, 6WD8, 6Y0G, 7K00, 7N1P, 7N2U, 7N2V, 7PJV, 7PJW, 7PJY, 7SSD, 7SSL, 8AM9, 8CAI, 8CEP, 8CF1 and 8CGU and the cryo-EM map that was used as a reference is available in the EM Data Bank under EMD-12573 (ref. ^30^). Data are available from the corresponding authors upon request. Source data are provided with this paper.
