# Supplementary material for: Paenilamicins are context-specific translocation inhibitors of protein synthesis
Source: Nat Chem Biol. 2024 Oct 17;20(12):1691–700. doi: 10.1038/s41589-024-01752-9 (PMC11581978; doi:10.1038/s41589-024-01752-9)
Supplement: Supplementary file 1 — Supplementary Figs. 1–5, Tables 1–3 and References. [file 41589_2024_1752_MOESM1_ESM.pdf]

nature chemical biology

Supplementary information https://doi.org/10.1038/s41589-024-01752-9

Paenilamicins are context-specific translocation inhibitors of protein synthesis

In the format provided by the authors and unedited

Table of Contents:

Title	Page
Supplementary Figure 1	2
Supplementary Figure 2	3
Supplementary Figure 3	4
Supplementary Figure 4	5
Supplementary Figure 5	6
Supplementary Table 1	7
Supplementary Table 2	8
Supplementary Table 3	9
Supplementary References	11

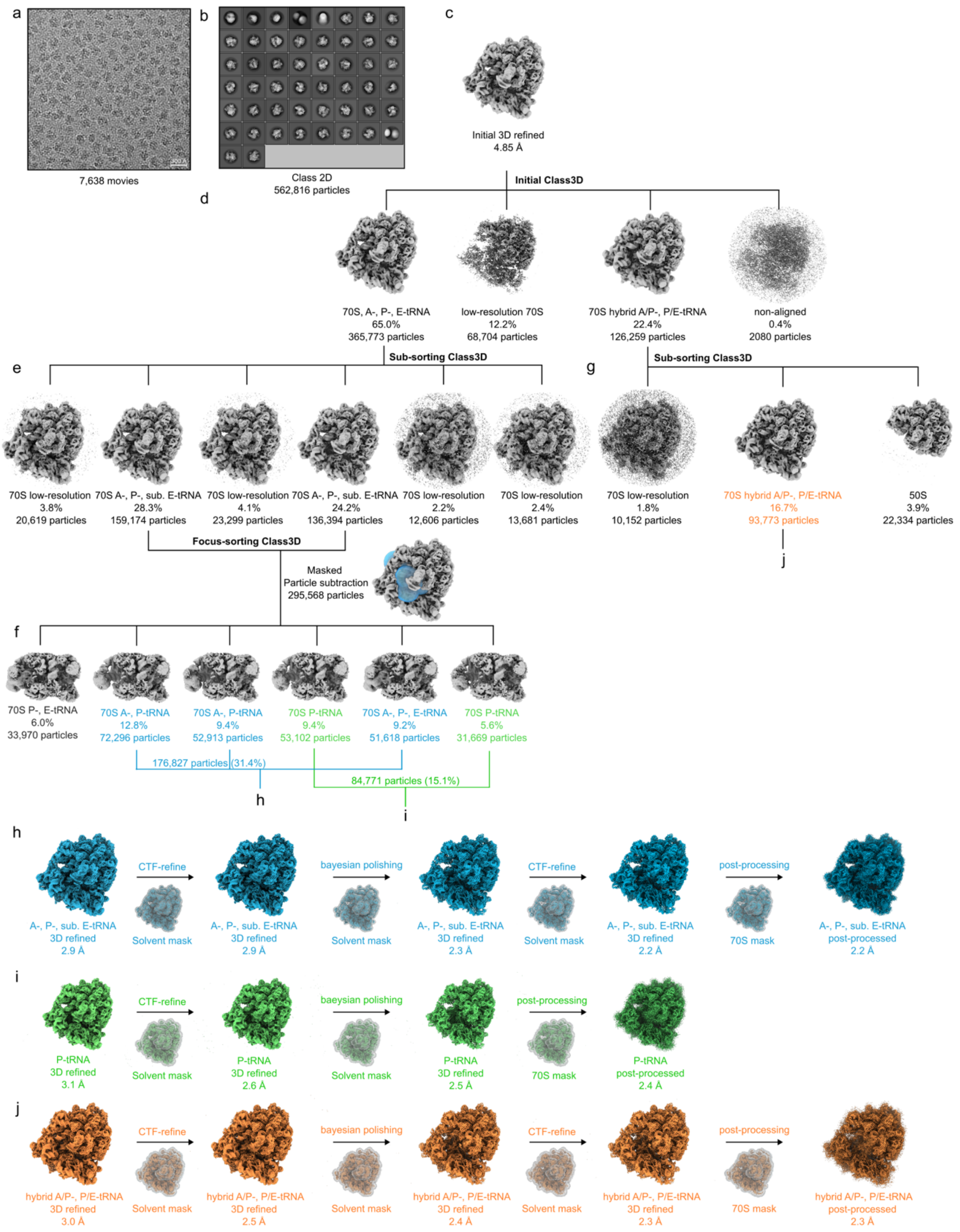

Supplementary Fig. 1: *In silico* sorting scheme of the *E. coli* 70S PamB2 complex. a-b, From 7,638 micrographs (**a**) a total of 562,816 ribosome-like particles were selected after 2D classification (**b**). **c**, Particles were subjected to an initial 3D refinement at 3x decimated pixel size. **d**, Particles were 3D classified for 100 iterations and resulted in four classes of which a non-rotated 70S class with A-, P- and E-site tRNAs (65.0%, 365,773 particles) and a rotated 70S with hybrid A/P- and P/E-tRNA (22.4%, 126,259 particles) were further sub-sorted. **e**, The non-rotated 70S class was 3D classified for 100 iterations and yielded six classes of which four classes were low resolution 70S particles and two classes of 70S with A-, P- and sub-stoichiometric E-site tRNA (52.5%, 295,568 particles) were combined. **f**, The combined classes from (**e**) were partially subtracted with a mask surrounding the tRNAs and 3D classified for 100 iterations yielding six classes. Classes containing A-, P- and E-site tRNA

(blue, 31.4%, 176,827 particles), as well as classes containing just P-site tRNA (green, 15.1%, 84,771 particles) were combined and further processed. **g**, The rotated 70S class was 3D classified for 100 iterations and yielded three classes which contained rotated 70S with hybrid A/P- and P/E-tRNA (orange, 16.7%, 93,773 particles), just 50S subunits (3.9%, 22,334 particles) and low resolution particles. The class containing rotated 70S was further processed. **h-j**, Particles were 3D refined at undecimated pixel size with a solvent mask, subjected to CTF refinement (4th order aberrations, anisotropic magnification and per-particle defocus value estimation), Bayesian polished, again CTF refined and after a final 3D refinement yielded (**h**) a final average resolution of 2.2 Å (at $FSC_{0.143}$) for the post-processed masked reconstruction of the non-rotated 70S complex containing A-, P- and sub. E-site tRNAs (blue), (**i**) a final average resolution of 2.4 Å (at $FSC_{0.143}$) for the post-processed masked reconstruction of the 70S complex containing P-tRNA (green) and (**j**) a final average resolution of 2.3 Å (at $FSC_{0.143}$) for the post-processed masked reconstruction of the rotated 70S complex containing hybrid A/P-, and P/E-tRNAs (orange). The single particles analysis was performed in RELION and a single time for 3D refinements, and employing the gold-standard, particles are randomly placed in one of two subsets and processed independently for both half-reconstructions. These subsets are maintained for CTF refinement.

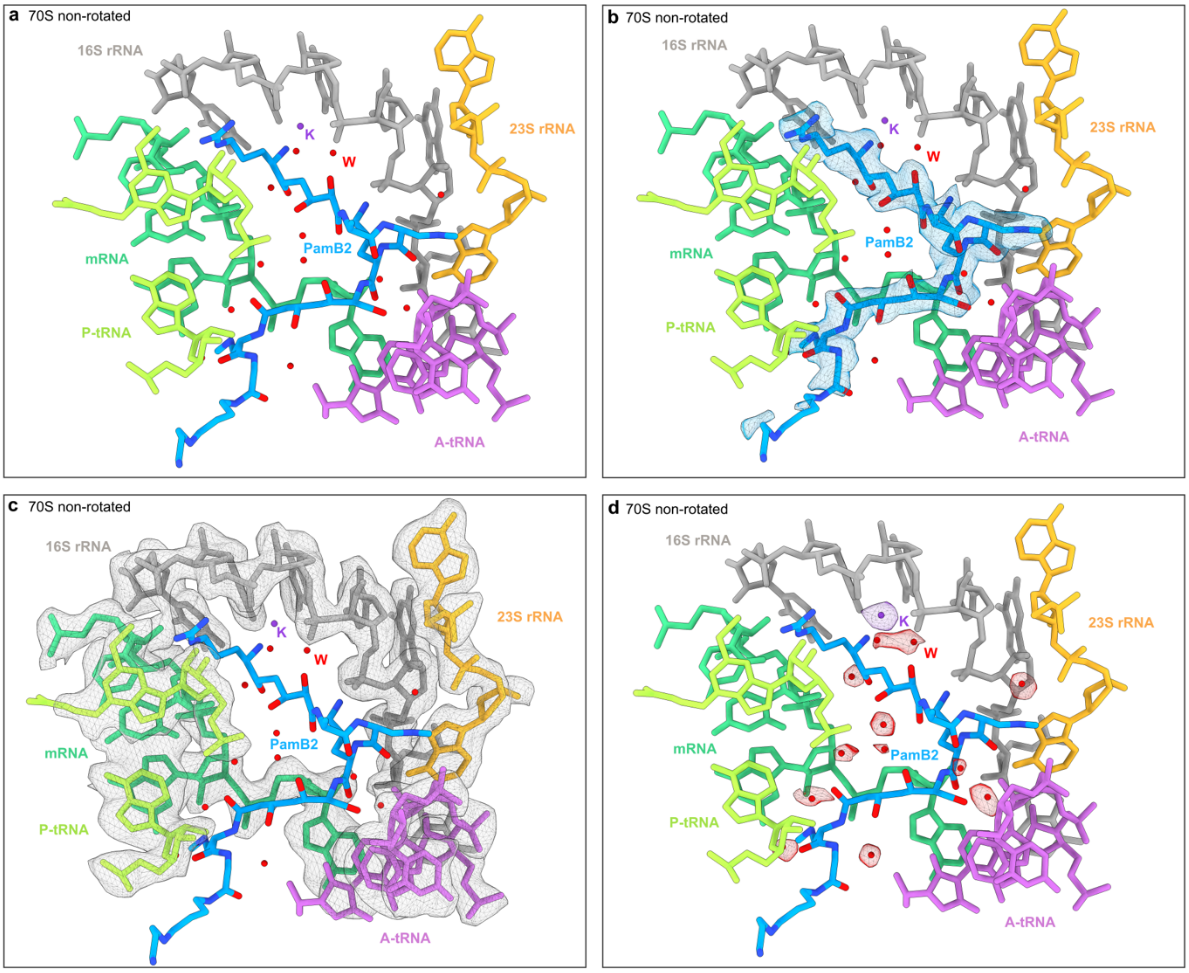

Supplementary Fig. 2: Cryo-EM density of the PamB2 binding pocket. a-d, PamB2 (blue) binding pocket surrounded by 16S rRNA nucleotides (grey), 23S rRNA nucleotides (yellow), mRNA (cyan), A- (purple) and P-site tRNA (light green), and waters (red) and a potassium ion (dark purple). Cryo-EM density of the non-rotated 70S PamB2 complex is shown as mesh for extracted density at one threshold for (**b**) PamB2, (**c**) surrounding nucleotides and (**d**) waters and potassium ion.

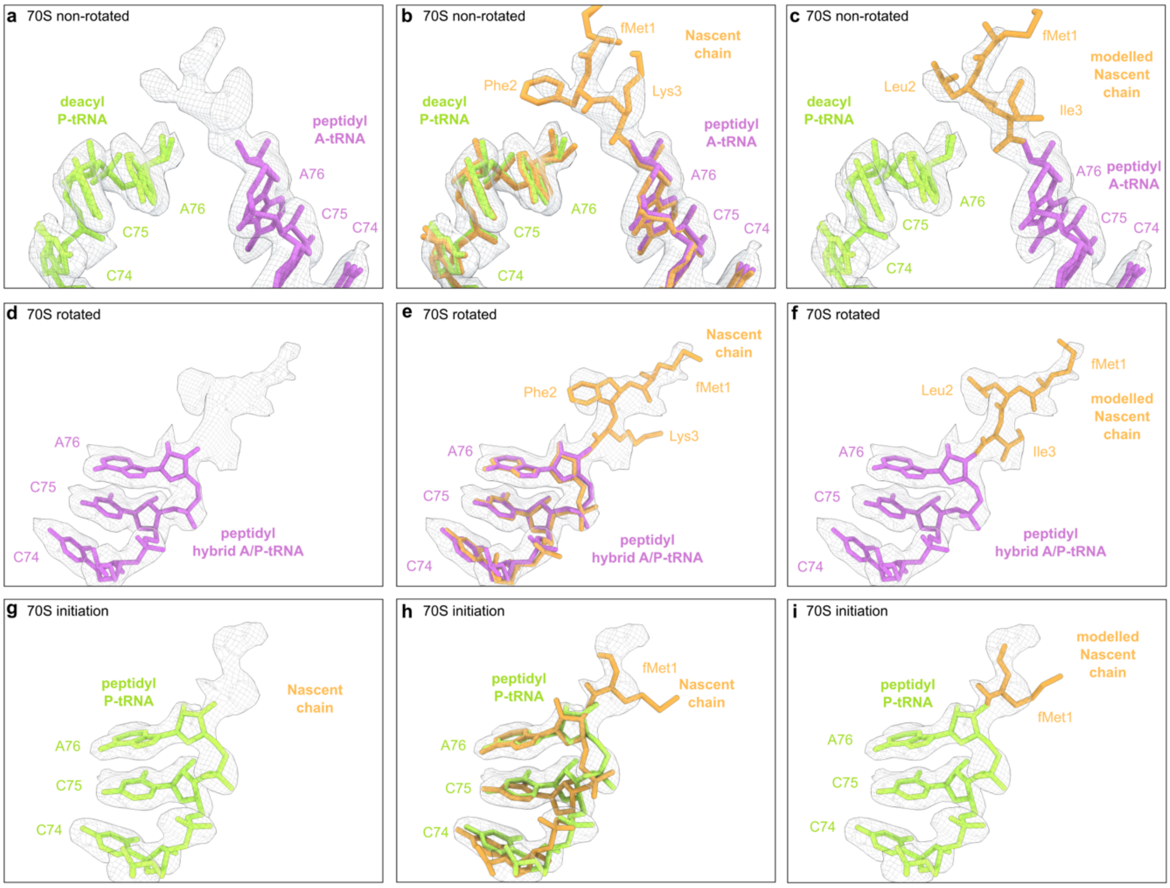

Supplementary Fig. 3: Additional cryo-EM density for the nascent chain attached to the CCA-end of the peptidyl-tRNA. a-i, Extracted cryo-EM densities of the respective complexes were shown as mesh with P-site tRNA (light green), A-site tRNA (purple) and hybrid A/P-site tRNA (purple). **a-c**, Additional cryo-EM density (**a**) on the A-site tRNA was superimposed with (**b**) a tri-peptide of a non-rotated 70S *E. coli* nascent chain from the PreC state (orange, PDB ID 7N1P)[1] and (**c**) a hypothetical molecular model of the fMet-Leu-Ile nascent chain connected to the A-site tRNA. **d-f**, Additional cryo-EM density (**d**) on the hybrid A/P-site tRNA was superimposed with (**e**) a tri-peptide of a rotated 70S *E. coli* nascent chain from the PreH1 state (orange, PDB ID 7N2U)[1] and (**f**) a hypothetical molecular model of the fMet-Leu-Ile nascent chain connected to the hybrid A/P-site tRNA. **g-h**, Additional cryo-EM density (**d**) on the P-site tRNA of the 70S initiation complex was superimposed with (**h**) an initiator fMet-tRNA^{fMet} from *an E. coli* 70S initiation state (orange, PDB ID 6WD0)[2] and (**f**) a hypothetical molecular model of the fMet nascent chain connected to the P-site tRNA.

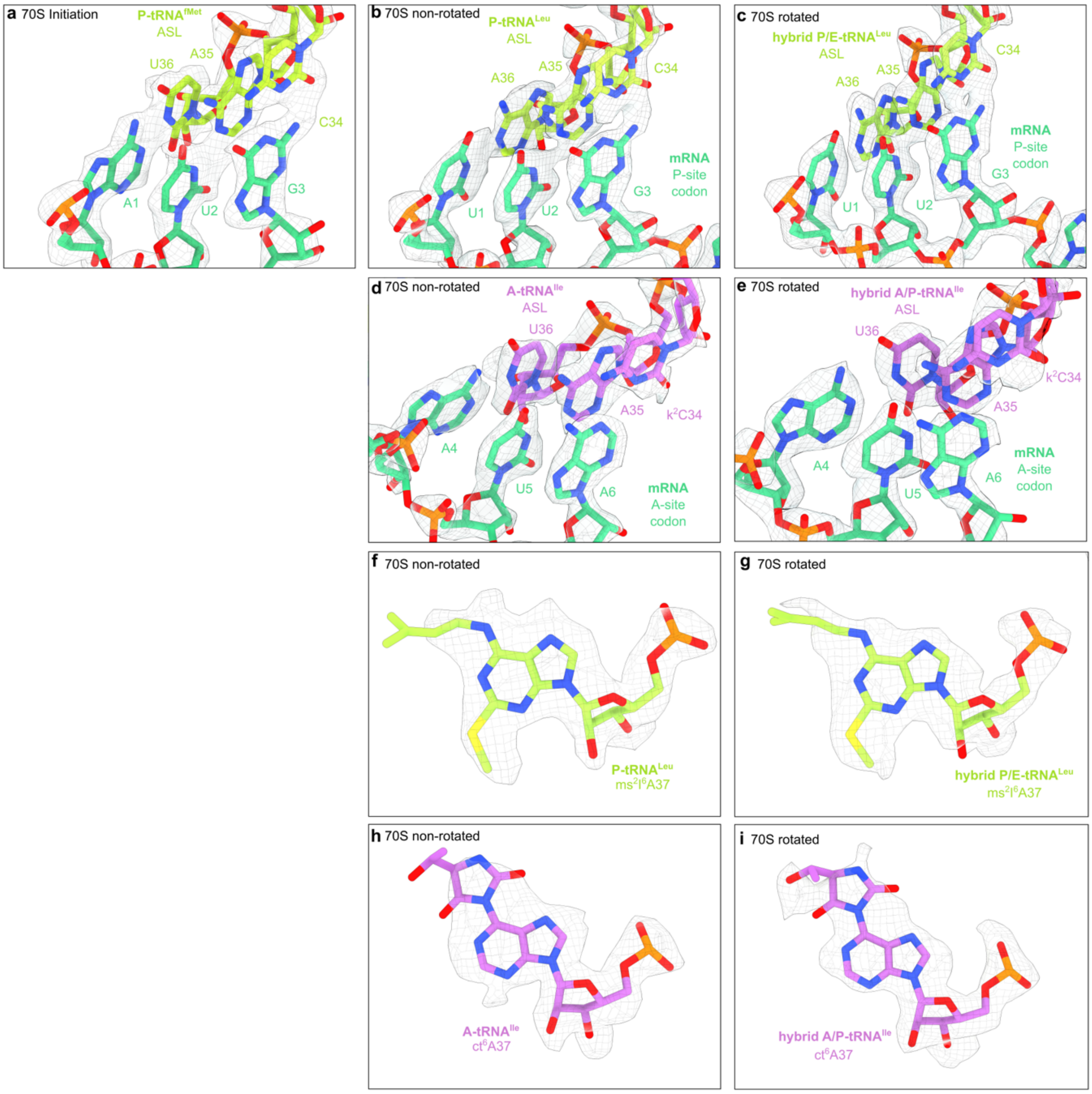

Supplementary Fig. 4: Codon-anticodon-interaction and tRNA identity. a-c, Extracted density of the codon-anticodon-interaction of the (**a**) P-site tRNA^{fMet} anticodon-stem loop (light green) and P-site codon (cyan, AUG) of 70S initiation complex, (**b**) P-site tRNA^{Leu} anticodon-stem loop (light green) and P-site codon (cyan, UUG) of the 70S non-rotated PamB2 complex and (**c**) hybrid P/E-tRNA^{Leu} anticodon-stem loop (light green) and P-site codon (cyan, UUG) of the 70S rotated PamB2 complex. **d-e**, Extracted density of the codon-anticodon-interaction of the (**d**) A-site tRNA^{Ile} anticodon-stem loop (purple) and A-site codon (cyan, AUA) of the 70S non-rotated complex and (**e**) hybrid A/P-tRNA^{Ile} anticodon-stem loop (purple) and P-site codon (cyan, UUG) of the 70S rotated PamB2 complex. **f-g**, Extracted cryo-EM density of the 2-methylhio-N6-isopentenyladenine (ms^{2}i^6, light green) modification at position 37 of the (**f**) P-site tRNA^{Leu} of the non-rotated PamB2 complex and the (**g**) hybrid P/E-site tRNA of the rotated PamB2 complex. **h-i**, Extracted cryo-EM density of the cyclic N6-threonylcarbamoyladenine (ct6, purple) modification at position 37 of the (**h**) A-site tRNA^{Ile} of the non-rotated PamB2 complex and the (**i**) hybrid A/P-site tRNA^{Ile} of the rotated PamB2 complex.

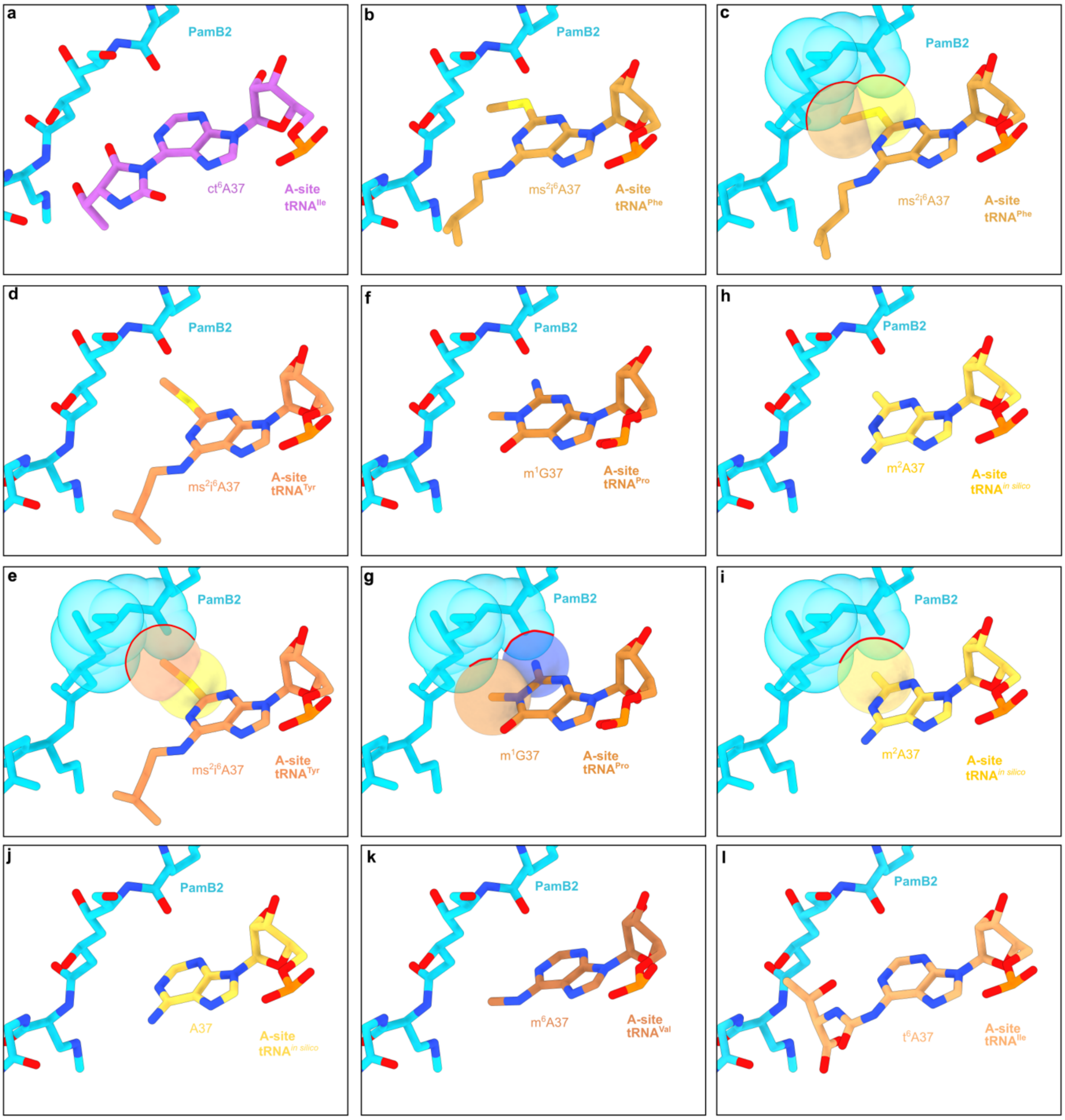

Supplementary Fig. 5: Influence of A37 modification of A-tRNA on PamB2 inhibition. a-l, PamB2 (light blue) from the non-rotated PamB2 complex superimposed with tRNAs with modified residues at position 37. **a**, The cyclic N6-threonylcarbamoyladenine (ct6, purple) modification at position 37 of the A-site tRNA^{Ile} from the non-rotated PamB2 complex. **b-c**, PamB2 from (**a**) superimposed with (**b**) tRNA^{Phe} with a 2-methylhio-N6-isopentenyladenine (ms^{2}i^{6}, light orange, PDB ID 1VY5)[3] at position 37 and sphere representation with (**c**) steric clashes highlighted in red. **d-e**, PamB2 from (**a**) superimposed with (**d**) tRNA^{Tyr} with a 2-methylhio-N6-isopentenyladenine (ms^{2}i^{6}, orange, PDB ID 4V8D)[4] at position 37 and sphere representation with (**e**) steric clashes highlighted in red. **f-g**, PamB2 from (**a**) superimposed with (**b**) tRNA^{Tyr} with a 1-methyl-guanine (m^{1}G, dark orange, PDB ID 6NUO)[5] at position 37 and sphere representation with (**g**) steric clashes highlighted in red. **h-i**, PamB2 from (**a**) superimposed with (**h**) an *in silico* modified tRNA with 2-methyl-adenine (m^{2}A, yellow) at position 37 and sphere representation with (**i**) steric clashes highlighted in red. **j,** PamB2 from (**a**) superimposed with an *in silico* tRNA with unmodified A37. **k**, PamB2 from (**a**) superimposed with tRNA^{Val} with 6-methyl-adenine (m^{6}A, brown, PDB ID 4V6Z)[6] in position 37. **i**, PamB2 from (**a**) superimposed with tRNA^{Ile} with N6-threonylcarbamyl (t^{6}A, beige, PDB ID 7N1P)[1] in position 37.

Supplementary Table 1. Cryo-EM data collection, modelling and refinement statistics.

	**Non-rotated 70S PamB2 complex** (EMD-18950) (PDB ID 8R6C)	**Rotated 70S PamB2 complex** (EMD-19004) (PDB ID 8R8M)	**70S Initiation complex** (EMD-50296) (PDB ID 9FBV)
**Data collection**			
Magnification (×)	96,000	96,000	96,000
Voltage (kV)	300	300	300
Electron exposure ($e^{-}/Å^{2}$)	60	60	60
Defocus range (μm)	-0.4 to -0.9	-0.4 to -0.9	-0.4 to -0.9
Pixel size (Å)	0.80	0.80	0.80
Symmetry imposed	C1	C1	C1
Initial particle images (no.)	562,816	562,816	562,816
Final particle images (no.)	176,827	93,773	84,771
Map resolution (Å)	2.2	2.3	2.4
FSC threshold	0.143	0.143	0.143
Map resolution range (Å)	1.9-3.2	2.0-3.5	2.1-4.0
**Refinement**			
Initial model used (PDB)	7K00	7K00	7K00
Model resolution (Å)	2.6	2.7	2.9
FSC threshold	0.5	0.5	0.5
Model resolution range (Å)	2.0-3.2	2.1-3.5	2.2-3.5
Map sharpening B factor ($Å^{2}$)	-4.74	-7.43	-8.04
Model composition			
Non-hydrogen atoms	145,011	141,381	140,097
Protein residues	5,585	5,573	5,585
RNA bases	4,547	4,530	4,470
B factors ($Å^{2}$)			
Protein	75.4	74.7	66.4
Nucleotide	64.9	61.0	54.8
R.M.S. deviations			
Bond lengths (Å)	0.010	0.010	0.010
Bond angles (°)	1.450	1.421	1.418
Validation			
MolProbity score	1.18	1.34	1.64
Clash score	0.68	0.78	1.92
Poor rotamers (%)	2.10	2.87	3.70
Ramachandran statistics			
Favoured (%)	96.64	96.34	96.20
Allowed (%)	3.05	3.29	3.58
Disallowed (%)	0.31	0.37	0.20

Supplementary Table 2. Primers used in this study.

Primer Name	Sequence (5’ -3’)
**ErmBL-UGA-NV1-R**	GGTTATAATGAATTTTGCTTATTAACGATAGAATTCTATCACTCAAATAGTAGATGTTTTATCTACATTACGCATTT
**T7-F**	TAATACGACTCACTATAGGG
**T7-ErmBL- F**	TAATACGACTCACTATAGGGAGACTTAAGTATAAGGAGGAAAAAATATGTTGGTATTCCAAATGCGTAATGTAGATAA
**UUG1 -F**	TTAGTATAAGGAGGAAAAAATATGTTGGTATTCCAAATGCGTAATG
**UUG2 -F**	TTAGTATAAGGAGGAAAAAATATGTTGTTGGTATTCCAAATGCGTAATG
**UUG3 -F**	TTAGTATAAGGAGGAAAAAATATGTTGTTGTTGGTATTCCAAATGCGTAATG
**UUG4 -F**	TTAGTATAAGGAGGAAAAAATATGTTGTTGTTGTTGGTATTCCAAATGCGTAATG
**UUG5 -F**	TTAGTATAAGGAGGAAAAAATATGTTGTTGTTGTTGTTGGTATTCCAAATGCGTAATG
**T7-SD-AUG-F**	TAATACGACTCACTATAGGGCTTAGTATAAGGAGGAAAAAATATG
**UUG4-UCU-F**	TTAGTATAAGGAGGAAAAAATATGTTGTTGTTGTTGTCTTTCCAAATGCGTAATGTAG
**UUG4-UCC-F**	TTAGTATAAGGAGGAAAAAATATGTTGTTGTTGTTGTCCTTCCAAATGCGTAATGTAG
**UUG4-UCA-F**	TTAGTATAAGGAGGAAAAAATATGTTGTTGTTGTTGTCATTCCAAATGCGTAATGTAG
**UUG4-UCG-F**	TTAGTATAAGGAGGAAAAAATATGTTGTTGTTGTTGTCGTTCCAAATGCGTAATGTAG
**NV1-ErmBL-R**	GGTTATAATGAATTTTGCTTATTAACGATAGAATTCTATCACTTACAAAATAGTAGATGTGATTTTATCTACATTACGCATTTGGAATAC
**NV1**	GGTTATAATGAATTTTGCTTATTAACC

Supplementary Table 3. mRNA templates used in this study.

Template Name	Sequence (5’ -3’)
**ErmBL**	TAATACGACTCACTATAGGGAGACTTAAGTATAAGGAGGAAAAAAT**ATG**TTGGTATTCCAAATGCGTAATGTAGATAAAACATCTACTATTTGAGTGATAGAATTCTATCGTTAATAAGCAAAATTCATTATAACC
**(UUG)2-ErmBL**	TAATACGACTCACTATAGGGCTTAGTATAAGGAGGAAAAAAT**ATG**TTGTTGGTATTCCAAATGCGTAATGTAGATAAAATCACATCTACTATTTTGTAAGTGATAGAATTCTATCGTTAATAAGCAAAATTCATTATAACC
**(UUG)$_3$-ErmBL**	TAATACGACTCACTATAGGGCTTAGTATAAGGAGGAAAAAAT**ATG**TTGTTGTTGGTATTCCAAATGCGTAATGTAGATAAAATCACATCTACTATTTTGTAAGTGATAGAATTCTATCGTTAATAAGCAAAATTCATTATAACC
**(UUG)$_4$-ErmBL**	TAATACGACTCACTATAGGGCTTAGTATAAGGAGGAAAAAAT**ATG**TTGTTGTTGTTGGTATTCCAAATGCGTAATGTAGATAAAATCACATCTACTATTTTGTAAGTGATAGAATTCTATCGTTAATAAGCAAAATTCATTATAACC
**(UUG)$_5$-ErmBL**	TAATACGACTCACTATAGGGCTTAGTATAAGGAGGAAAAAAT**ATG**TTGTTGTTGTTGTTGGTATTCCAAATGCGTAATGTAGATAAAATCACATCTACTATTTTGTAAGTGATAGAATTCTATCGTTAATAAGCAAAATTCATTATAACC
**(UUG)$_4$-UCU-ErmBL**	TAATACGACTCACTATAGGGCTTAGTATAAGGAGGAAAAAAT**ATG**TTGTTGTTGTTGTCTTTCCAAATGCGTAATGTAGATAAAATCACATCTACTATTTTGTAAGTGATAGAATTCTATCGTTAATAAGCAAAATTCATTATAACC
**(UUG)$_4$-UCC-ErmBL**	TAATACGACTCACTATAGGGCTTAGTATAAGGAGGAAAAAAT**ATG**TTGTTGTTGTTGTCCTTCCAAATGCGTAATGTAGATAAAATCACATCTACTATTTTGTAAGTGATAGAATTCTATCGTTAATAAGCAAAATTCATTATAACC
**(UUG)$_4$-UCA-ErmBL**	TAATACGACTCACTATAGGGCTTAGTATAAGGAGGAAAAAAT**ATG**TTGTTGTTGTTGTCATTCCAAATGCGTAATGTAGATAAAATCACATCTACTATTTTGTAAGTGATAGAATTCTATCGTTAATAAGCAAAATTCATTATAACC
**(UUG)$_4$-UCG-ErmBL**	TAATACGACTCACTATAGGGCTTAGTATAAGGAGGAAAAAAT**ATG**TTGTTGTTGTTGTCGTTCCAAATGCGTAATGTAGATAAAATCACATCTACTATTTTGTAAGTGATAGAATTCTATCGTTAATAAGCAAAATTCATTATAACC
**ErmBL-ATG**	TAATACGACTCACTATAGGGAGACTTAAGTATAAGGAGGAAAAAAT**ATG**ATGGTATTCCAAATGCGTAATGTAGATAAAACATCTACTATTTGAGTGATAGAATTCTATCGTTAATAAGCAAAATTCATTATAACC
**ErmBL-CTG**	TAATACGACTCACTATAGGGAGACTTAAGTATAAGGAGGAAAAAAT**ATG**CTGGTATTCCAAATGCGTAATGTAGATAAAACATCTACTATTTGAGTGATAGAATTCTATCGTTAATAAGCAAAATTCATTATAACC
**ErmBL-GTG**	TAATACGACTCACTATAGGGAGACTTAAGTATAAGGAGGAAAAAAT**ATG**GTGGTATTCCAAATGCGTAATGTAGATAAAACATCTACTATTTGAGTGATAGAATTCTATCGTTAATAAGCAAAATTCATTATAACC
**MLIF-UAA**	TAATACGACTCACTATAGGGAGACTTAAGTATAAGGAGGAAAAAAT**ATG**TTGATATTCTAAATGCGTAATGTAGATAAAACATCTACTATTTAAGTGATAGAATTCTATCGTTAATAAGCAAAATTCATTATAACC

**MFKAFK**	ATTAATACGACTCACTATAGGGCAACCTAAAACTTACACACGCC CCGGTAAGGAAATAAAA**ATG**TTCAAAGCATTCAAAAACATCATAC GTACTCGTACTCTTTAAGCGCAGGCAAGGTTAATAAGCAAAATT CATTATAACC

Supplementary References

1 Rundlet, E. J. *et al.* Structural basis of early translocation events on the ribosome. *Nature* **595**, 741-745 (2021).

2 Loveland, A. B., Demo, G. & Korostelev, A. A. Cryo-EM of elongating ribosome with EF-Tu*GTP elucidates tRNA proofreading. *Nature* **584**, 640-645 (2020).

3 Polikanov, Y. S., Steitz, T. A. & Innis, C. A. A proton wire to couple aminoacyl-tRNA accommodation and peptide-bond formation on the ribosome. *Nat Struct Mol Biol* **21**, 787-793 (2014).

4 Demeshkina, N., Jenner, L., Westhof, E., Yusupov, M. & Yusupova, G. A new understanding of the decoding principle on the ribosome. *Nature* **484**, 256-259 (2012).

5 Hoffer, E. D. *et al.* Structural insights into mRNA reading frame regulation by tRNA modification and slippery codon-anticodon pairing. *eLife* **9** (2020).

6 Bock, L. V. *et al.* Energy barriers and driving forces in tRNA translocation through the ribosome. *Nat Struct Mol Biol* **20**, 1390-1396 (2013).
