# Supplementary material for: Paenilamicins are context-specific translocation inhibitors of protein synthesis
Source: Nat Chem Biol. 2024 Oct 17;20(12):1691–700. doi: 10.1038/s41589-024-01752-9 (PMC11581978; doi:10.1038/s41589-024-01752-9)
Supplement: Supplementary file 2 — Reporting Summary [file 41589_2024_1752_MOESM2_ESM.pdf]

nature portfolio

Corresponding author(s): Wilson DN

Last updated by author(s): Jul 17, 2024

Reporting Summary

Nature Portfolio wishes to improve the reproducibility of the work that we publish. This form provides structure for consistency and transparency in reporting. For further information on Nature Portfolio policies, see our Editorial Policies and the Editorial Policy Checklist.

Statistics

For all statistical analyses, confirm that the following items are present in the figure legend, table legend, main text, or Methods section.

n/a	Confirmed	
☐	☒	The exact sample size (*n*) for each experimental group/condition, given as a discrete number and unit of measurement
☐	☒	A statement on whether measurements were taken from distinct samples or whether the same sample was measured repeatedly
☒	☐	The statistical test(s) used AND whether they are one- or two-sided<br>*Only common tests should be described solely by name; describe more complex techniques in the Methods section.*
☒	☐	A description of all covariates tested
☒	☐	A description of any assumptions or corrections, such as tests of normality and adjustment for multiple comparisons
☐	☒	A full description of the statistical parameters including central tendency (e.g. means) or other basic estimates (e.g. regression coefficient) AND variation (e.g. standard deviation) or associated estimates of uncertainty (e.g. confidence intervals)
☒	☐	For null hypothesis testing, the test statistic (e.g. *F*, *t*, *r*) with confidence intervals, effect sizes, degrees of freedom and *P* value noted<br>*Give P values as exact values whenever suitable.*
☒	☐	For Bayesian analysis, information on the choice of priors and Markov chain Monte Carlo settings
☒	☐	For hierarchical and complex designs, identification of the appropriate level for tests and full reporting of outcomes
☒	☐	Estimates of effect sizes (e.g. Cohen's *d*, Pearson's *r*), indicating how they were calculated

Our web collection on statistics for biologists contains articles on many of the points above.

Software and code

Policy information about availability of computer code

Data collection	CryoEM data were collected using the EPU 2.6.1 software (FEI, Netherlands)
Data analysis	RELION v4.0.1 with MotionCor2 v1.2.1, CTFFIND 4.1.14, and crYOLO v1.8.04b47 were used for processing micrographs, picking particles, classification and refining cryo-EM maps. BSoft 2.1.1 was used to calculate local resolution. Coot v0.9.8.92 and aceDRG (CCP4 v8.0.017) for model building and Refmac 5 in Servalcat v0.4.28 and Phenix (1.20.1-4487) for model refinement and statistics. Figures were generated using ChimeraX v1.6.1 and Inkscape 1.3

For manuscripts utilizing custom algorithms or software that are central to the research but not yet described in published literature, software must be made available to editors and reviewers. We strongly encourage code deposition in a community repository (e.g. GitHub). See the Nature Portfolio guidelines for submitting code & software for further information.

Data

Policy information about availability of data

All manuscripts must include a data availability statement. This statement should provide the following information, where applicable:

- Accession codes, unique identifiers, or web links for publicly available datasets
- A description of any restrictions on data availability
- For clinical datasets or third party data, please ensure that the statement adheres to our policy

Micrographs have been deposited as uncorrected frames in the Electron Microscopy Public Image Archive (EMPIAR) with the accession codes EMPIAR-12080 [https://www.ebi.ac.uk/pdbe/emdb/empiar/entry/12080]. Cryo-EM maps have been deposited in the Electron Microscopy Data Bank (EMDB) with accession codes EMD-18950 [https://www.ebi.ac.uk/pdbe/entry/emdb/EMD-18950] (Non-rotated 70S PamB2 complex), EMD-19004 [https://www.ebi.ac.uk/pdbe/entry/emdb/EMD-19004] (Rotated 70S PamB2 complex), and EMD-50296 [https://www.ebi.ac.uk/pdbe/entry/emdb/EMD-50296] (Initiation 70S complex). Molecular models

have been deposited in the Protein Data Bank with accession codes 8R6C [https://doi.org/10.2210/pdb8R6C/pdb] (Non-rotated 70S PamB2 complex), 8R8M [https://doi.org/10.2210/pdb8R8M/pdb] (Rotated 70S PamB2 complex), 9FBV [https://doi.org/10.2210/pdb9FBV/pdb] (Initiation 70S complex). Structures from prior studies were used in this work for comparison, alignments and for modelling and are available in the Protein Data Bank, with PDB ID 1I95, 1VY4, 1VY5, 4V6Z, 4V8D, 4W2I, 4W2H, 6NUO, 6WD0, 6WD2, 6WD8, 6Y0G, 7K00, 7N1P, 7N2U, 7N2V, 7PJV, 7PJW, 7PJY, 7SSD, 7SSL, 8AM9, 8CAI, 8CEP, 8CF1, 8CGU. Source data are provided with this paper. The cryo-EM map that was used as reference is available in the EM Data Bank, with EMD-12573.

Field-specific reporting

Please select the one below that is the best fit for your research. If you are not sure, read the appropriate sections before making your selection.

☒ Life sciences ☐ Behavioural & social sciences ☐ Ecological, evolutionary & environmental sciences

For a reference copy of the document with all sections, see nature.com/documents/nr-reporting-summary-flat.pdf

Life sciences study design

All studies must disclose on these points even when the disclosure is negative.

Sample size	No statistical methods were used to determine sample size. The sample size (particle number) for the cryoEM dataset was chosen based on previous experience of the number of particles necessary to obtain high resolution of the final complexes.
Data exclusions	Micrographs with low estimated resolution or poorly fitted CTFs were discarded, as were particles that clustered into poorly defined classes during 2D and 3D classification.
Replication	Toeprinting experiments were done in duplicate and were successful and are presented in ED Fig 2, Fig. 3b, Fig. 4a and 4b and Fig 5c. Duplicates gels can be found in the Source Data. In vitro translation and cytotoxicity assays shown in Fig 5b and c, respectively, were done in triplicate and replication was successful.
Randomization	For 3D refinement in RELION, particles are randomly placed in one of two subsets. These subsets are maintained for CTF refinement. Otherwise, no randomization was performed because they did not require randmization.
Blinding	Blinding was not relevant because all data were discrete and/or raw data is reported in the manuscript.

Reporting for specific materials, systems and methods

We require information from authors about some types of materials, experimental systems and methods used in many studies. Here, indicate whether each material, system or method listed is relevant to your study. If you are not sure if a list item applies to your research, read the appropriate section before selecting a response.

Materials & experimental systems

n/a	Involved in the study	
☒	☐	Antibodies
☐	☒	Eukaryotic cell lines
☒	☐	Palaeontology and archaeology
☒	☐	Animals and other organisms
☒	☐	Human research participants
☒	☐	Clinical data
☒	☐	Dual use research of concern

Methods

n/a	Involved in the study	
☒	☐	ChIP-seq
☒	☐	Flow cytometry
☒	☐	MRI-based neuroimaging

Eukaryotic cell lines

Policy information about cell lines

Cell line source(s)	The NIH/3T3 murine fibroblas cell line was purchased from the American Type Culture Collection (ATCC, cat #: CRL-1658).
Authentication	The cell line was not authenticated, except visually using light microscopy
Mycoplasma contamination	The cell line was not tested for mycoplasma.
Commonly misidentified lines (See ICLAC register)	No commonly misidentified cell lines were used in this study.
